# Identification of tumor-related genes via RNA sequencing of tumor tissues in* Xenopus tropicalis*

**DOI:** 10.1038/s41598-023-40193-7

**Published:** 2023-08-14

**Authors:** Kazuki Kitamura, Takayoshi Yamamoto, Haruki Ochi, Makoto Suzuki, Nanoka Suzuki, Takeshi Igawa, Tadashi Yoshida, Mitsuru Futakuchi, Hajime Ogino, Tatsuo Michiue

**Affiliations:** 1 Department of Life Sciences, Graduate School of Arts and Sciences, The University of Tokyo, 3-8-1 Komaba, Meguro-Ku, Tokyo, 153-8902 Japan; 2 Institute for Promotion of Medical Science Research, Faculty of Medicine, Yamagata University, 2-2-2 Iida-Nishi, Yamagata City, Yamagata 990-9585 Japan; 3 Amphibian Research Center, Hiroshima University, 1-3-2 Kagamiyama, Higashi-Hiroshima City, Hiroshima 739-8511 Japan; 4 Department of Pathology, Faculty of Medicine, Yamagata University, 2-2-2 Iida-Nishi, Yamagata City, Yamagata 990-9585 Japan; 5 Department of Biological Sciences, Graduate School of Science, The University of Tokyo, 7-3-1 Hongo, Bunkyo-Ku, Tokyo, 113-0033 Japan

**Keywords:** Oncogenes, Tumour biomarkers, Transcriptomics, Model vertebrates

## Abstract

Cancer treatment is still challenging because the disease is often caused by multiple mutations. Although genomic studies have identified many oncogenes and tumor suppressor genes, gene sets involved in tumorigenesis remain poorly understood. *Xenopus*, a genus of aquatic frogs, is a useful model to identify gene sets because it can be genetically and experimentally analyzed. Here, we analyzed gene expression in tumor tissues of three individuals in *Xenopus tropicalis* and identified 55 differentially expressed genes (DEGs). Gene ontology (GO) analysis showed that the upregulated genes in the tumor tissues were enriched in GO terms related to the extracellular matrix and collagen fibril organization. Hierarchical clustering showed that the gene expression patterns of tumor tissues in *X. tropicalis* were comparable to those of human connective, soft, and subcutaneous tissue-derived cancers. Additionally, pathway analysis revealed that these DEGs were associated with multiple pathways, including the extracellular matrix, collagen fibril organization, MET signaling, and keratan sulfate. We also found that the expression tendency of some DEGs that have not been well analyzed in the cancer field clearly determines the prognosis of human cancer patients. This study provides a remarkable reference for future experimental work on *X. tropicalis* to identify gene sets involved in human cancer.

## Introduction

Gene mutations occur at a certain frequency. For instance, they are caused by environmental cues, including smoking, alcohol consumption, and dietary habits^1–3^. The accumulation of genetic mutations causes various diseases, among which cancer is highly common^4^. It is estimated that approximately half of all humans develop cancer during their lifetime, and the early detection of cancer is particularly crucial for reducing the risk of death. Therefore, genetic testing has gained increasing attention in cancer treatment.

Recently, many disease-associated genes have been identified using next-generation sequencing (NGS). For example, 736 genes have been defined as “cancer-driving genes” by the Cancer Gene Census (CGC) (version 97, November 2022), which is a key resource within the Catalogue of Somatic Mutations in Cancer (COSMIC), comprising a long-term, ongoing effort to catalog and describe all genes with causal impact in human cancer^5^. For instance, in breast cancer, BRCA1/2 genes are used for genetic testing, and for patients with mutations in these genes, treatments such as mastectomy are performed even prior to the onset of cancer progression^6^. However, in many cases, a single mutation is insufficient to induce cancer, i.e., the accumulation of gene mutations, rather than a single gene mutation, causes cancer^7,8^. Nevertheless, the gene sets critical for cancer development and progression are not yet well determined. Therefore, determining these gene sets enables diagnosis and treatment before the onset of cancer progression, thereby reducing the risk of cancer-related deaths.

Many genes exhibit differences in expression levels between normal and cancer tissues. In addition, certain genes demonstrate significant changes in expression levels in cancer tissues with a poor prognosis^9,10^. It is possible that subsequent treatment could be changed or modified depending on the gene expression pattern in the cancer. Thus, in cancer research, not only mutation analysis but also expression analysis is important. However, although gene expression levels have been just analyzed for tumor tissues, studies linked the expression analysis to cancer treatment are limited.

Genetic and experimental analyses are required to determine gene sets associated with cancer. In this regard, genome-editing technologies, such as the CRISPR/Cas9 system, are useful^11^. Most of these screenings have been performed using cultured cells. However, gene expressions in vitro differ from those *in vivo*^12^. Thus, there is still a gap between in vivo and in vitro screening, and in vivo screening is crucial for biomedical research. Because in vivo assays in humans are difficult due to ethical dilemmas, useful model organisms are required.

*Xenopus tropicalis* (*X. tropicalis*), a genus of aquatic frogs, has a diploid genome, rendering it an ideal organism for genome-editing experiments^13^. Recently, many tumors have been found across several generations of the Nigerian H (NH) strain of *X. tropicalis*. In this study, we performed RNA-seq analysis of three *X. tropicalis* individuals of the NH strain with tumors to identify tumorigenic genes and compared them with human cancer tissues to provide novel responsible genes for oncogenesis.

## Results

### Observation of *X. tropicalis* tumor tissues

**Figure 1:**
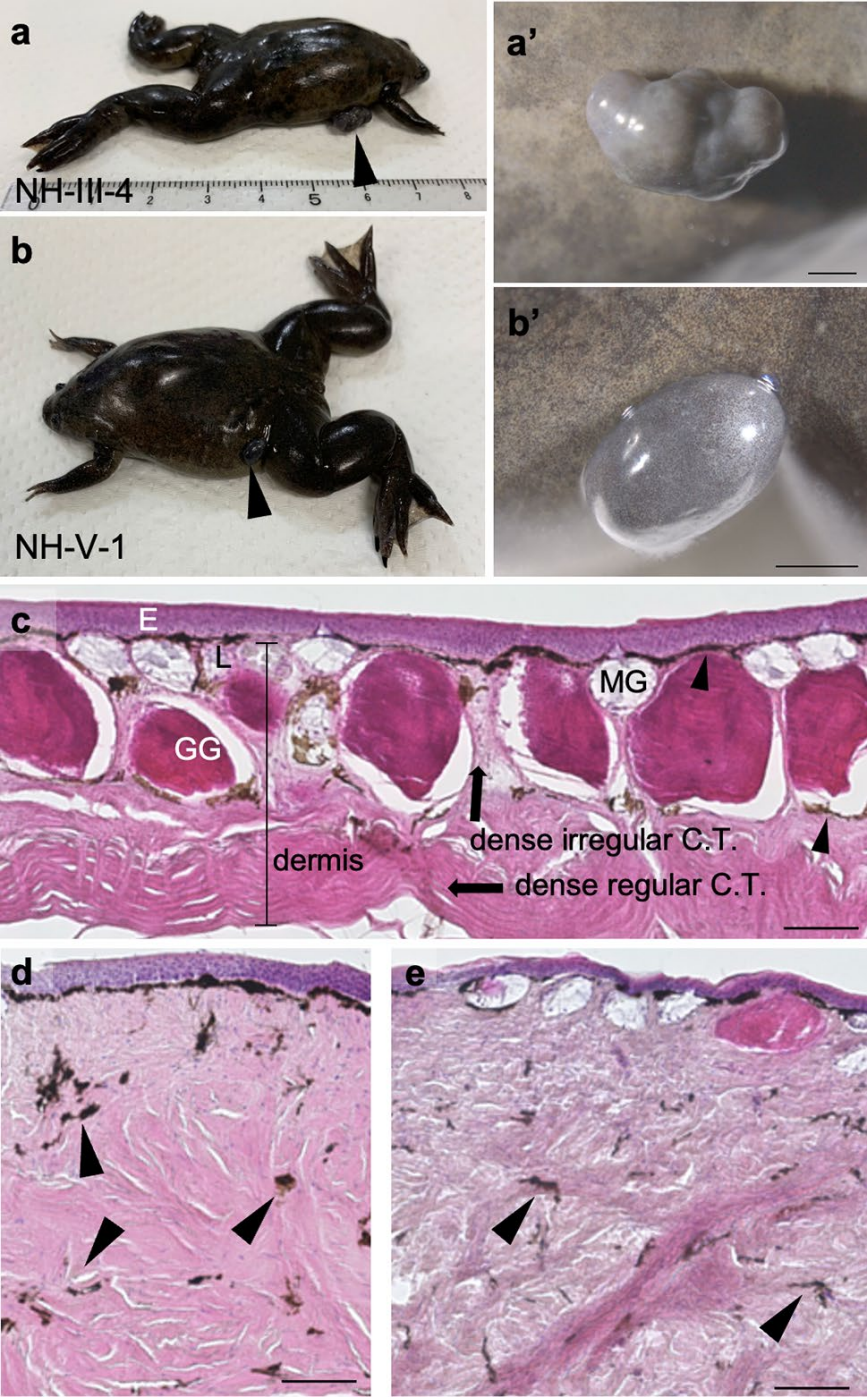
External view and histological analysis of *X. tropicalis* tumors. (**a,b**) External view of NH-III-4 and NH-V-1 tumor individuals. Arrowheads indicate the tumors. (**a’,b’**) Enlarged view of the tumors. Scale bar indicates 2 mm (**a’,b’**). (**c**) Histological analysis of normal tissue, stained by HE. Skin is composed of the epidermis (E) and dermis, which is subdivided into the dense regular connective tissue (C.T.) (arrow), dense irregular C.T. (arrow) and loose C.T. (L). Arrowheads indicate the pigment. The pigment adjacent to the epidermis is commonly referred to as the chromatophore. GG, granular gland; MG, mucous gland. (**d,e**) Histological analysis of tumors of NH-III-4 (**d**) and NH-V-1 (**e**). Arrowheads in (**d,e**) indicate the pigment. Scale bar indicates 100 μm (**c–e**).

Tumors developed spontaneously over several generations in the NH strain (Fig. S1), one of which (NH-VIII-7) has already been histologically analyzed^14^. In this study, we analyzed the gene expression profiles of three tumor-bearing individuals, including NH-VIII-7. Similar to NH-VIII-7, tumors were found in the flanks of NH-III-4 and NH-V-1 (Fig. 1a,b). The tumors were hard and marginally different in color from the surrounding skin tissue, i.e., purple, gray, or reddish brown, and had a dome shape (Fig. 1a',b').

For further detailed observation, the tissues were sectioned and stained with Hematoxylin and Eosin (HE). In normal tissues, the granules and glands are localized just inside the epidermis. The dermis, which is derived from the mesoderm, is layered under the granules and glands (Fig. 1c). In the tumor tissue, the mucous glands and serous glands were missing in NH-III-4 (Fig. 1d). Instead of this, connective tissue (C.T.) of the dermis seemed to have increased, particularly in dense regular C.T. of the dermis. Similar to this, in NH-V-1 (Fig. 1e), the dermis showed remarkable thickening. In addition, pigments were scattered in the tumor tissues. These observations suggest that these individuals are considered to have defects mainly in the mesoderm-derived tissues.

### Differences in gene expression patterns among samples using hierarchical clustering

We performed RNA-seq analysis to investigate the gene expression patterns of tumor tissues (Tumor Tissue (flank) #1-#3, defined as TT-body1-3 hereafter). We also collected skin tissues, which have no tumors, from the legs of three tumor-bearing individuals (non-tumor tissue (leg) in Tumor-bearing individual #1-#3, TI-leg1-3) and the flank and leg of three no-tumor-bearing individuals (non-tumor tissue (flank) in No-tumor-bearing individual #1-#3, NI-body1-3; non-tumor tissue (leg) in No-tumor-bearing individual #1-#3, NI-leg1-3), as negative control samples (Table 1).

**Table 1:** Sample names of *Xenopus* tumor tissues.

Region	Tumor-bearing individual	No-tumor-bearing individual
Flank	Tumor tissue:** TT-body**	Non tumor tissue:** NI-body**
Leg	Non tumor tissue: **TI-leg**	Non tumor tissue: **NI-leg**

**Figure 2:**
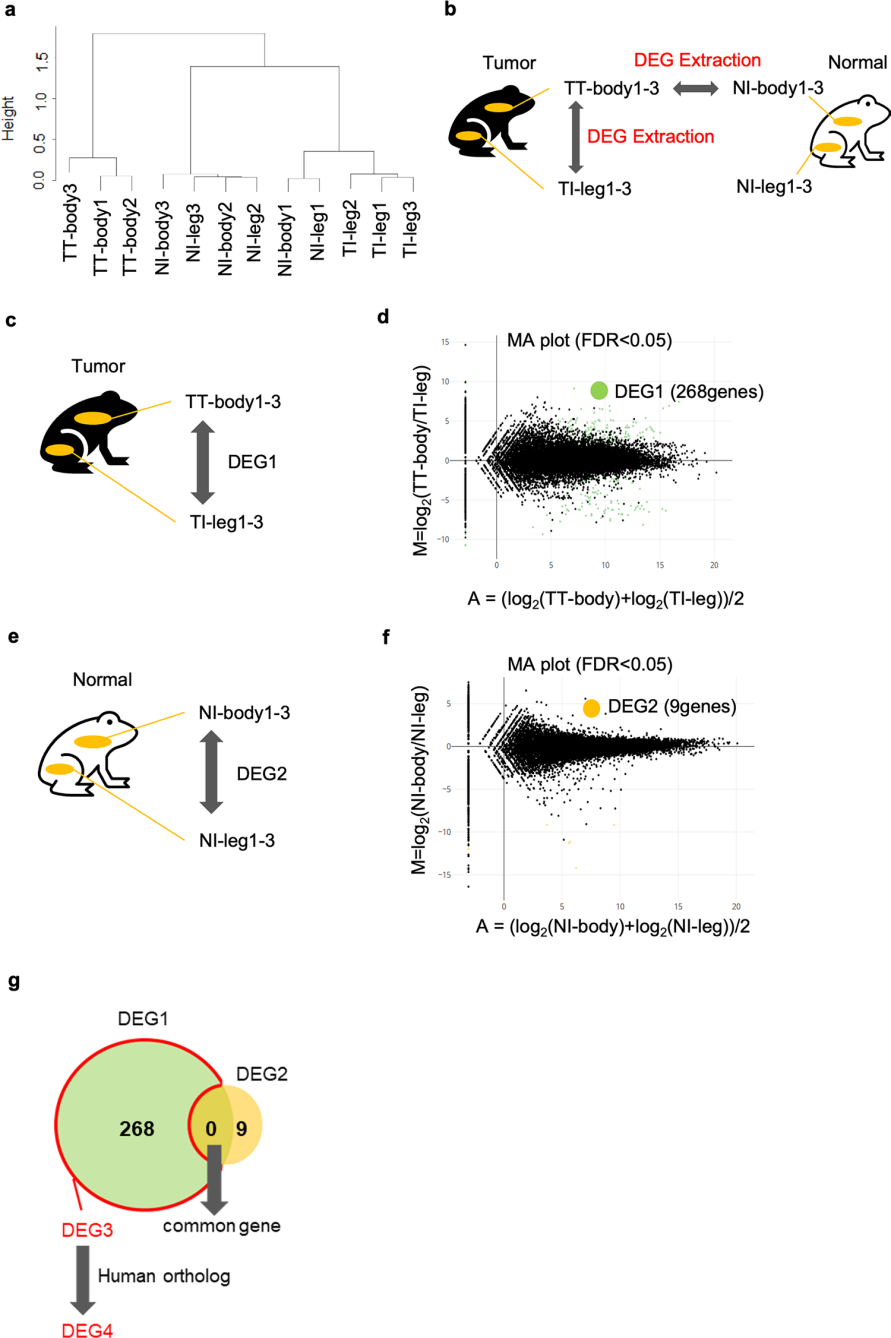
Hierarchical clustering of all analyzed tumors in *Xenopus* and DEGs of TT-body1-3 vs TI-leg1-3. (**a**) Hierarchical clustering of 12 analyzed samples. Difference of the expression patten among the samples was indicated with a tree diagram. (**b**) Schematic diagram of extraction of differentially expressed genes (DEGs) in *Xenopus*, which we analyzed in this study. (**c,d**) Schematic diagram of DEGs extraction (TT-body1-3 vs TI-leg1-3) (**c**) and the MA plot (**d**). Green dots indicate DEGs with statistical significance (FDR < 0.05) in (**d**). (**e,f**) Schematic diagram of DEGs extraction (NI-body1-3 vs NI-leg1-3) (**e**), and the MA plot (**f**). Orange dots indicate DEGs with statistical significance (FDR < 0.05) in (**f**). (**g**) Venn diagram showing DEG3. DEG3 is DEG1—DEG1 ∩ DEG2.

To identify differences in gene expression patterns among samples, we performed hierarchical clustering using Pearson’s correlation coefficients^15^ (Fig. 2a, Fig. S2a). The three tumor tissues were positioned close to each other in the dendrogram, suggesting that their gene expression patterns were similar. Because the results of both hierarchical clustering and histological analysis showed that the three samples were similar, TT-body1-3 were analyzed as biological replicates in subsequent analyses.

In addition, because the flank and leg skin tissues of no-tumor-bearing individuals were also closely positioned in the dendrogram (Fig. 2a), the expression patterns are similar independent to the region, flank or leg, of the skin. Thus, we used the leg skin of tumor-bearing individuals as a control sample. However, the leg skins possibly show abnormal gene expression patterns. Therefore, both the leg skin of tumor-bearing individuals and the flank skin of no-tumor-bearing individuals were used as control samples for subsequent analyses (Fig. 2b).

### Analysis of differential gene expression between the flank skin (tumor) and the leg skin (non-tumor) in tumor-bearing individuals

To identify differentially expressed genes (DEGs), we compared gene expression between TT-body1-3 and TI-leg1-3 (Fig. 2c) with Trimmed Means of M values (TMM) method^16^. We identified 268 DEGs (DEG1; Supplementary Table 1) (FDR < 0.05) and visualized the gene expression differences using MA plot (Fig. 2d). DEG1 possibly includes DEGs due to region difference, flank or leg. To only detect differences in gene expression due to tumorigenesis, we decided to exclude the DEGs of NI-leg1-3 and NI-body1-3 from DEG1 (Fig. 2e). 9 genes were output as DEGs (DEG2; Fig. 2f, Supplementary Table 2). We thought of removing the common genes of both DEG1 and DEG2, which were considered region-independent genes, that is, tumor-specific genes (DEG3) (Fig. 2g) but, the number of common genes was zero. So, in this case, DEG1 was identical to DEG3 (Fig. 1g, Supplementary Table 1).

**Figure 3:**
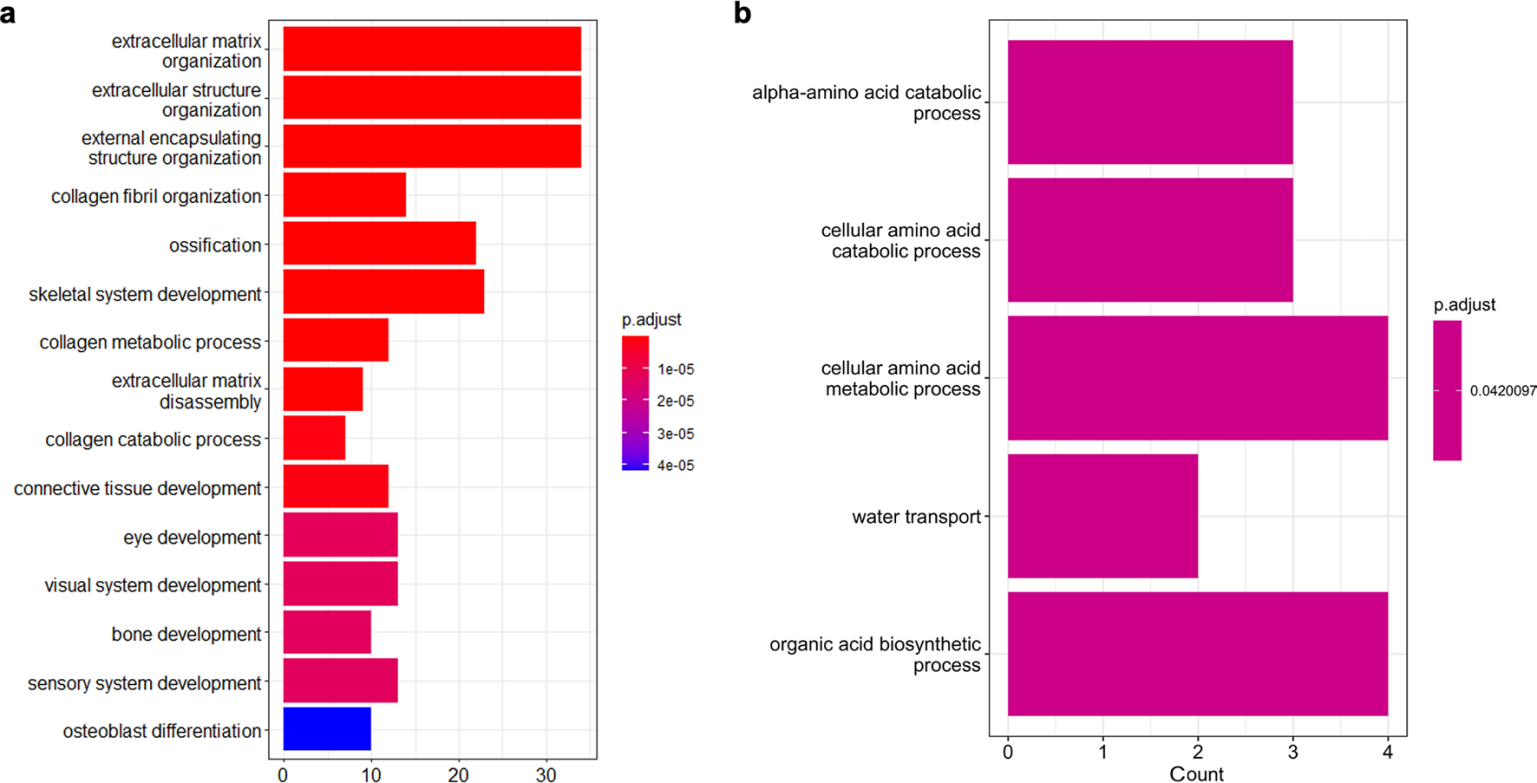
GO analysis of up- or down-regulated genes of DEG4. GO analysis of upregulated genes (**a**) or downregulated genes (**b**). Bar plots represents number of genes involved in each term. Adjusted p-value was represented by color scale, and the statistically significance level decreased from red (higher significance) to blue (lower significance).

Gene Ontology (GO) enrichment analysis was performed using DEG3, referred to the human GO annotation file. Human orthologous genes were determined by the Reciprocal best BLAST Hit (RBH) using OrthoFinder^17,18^. The number of genes that DEG3 finally converted to human genes was 138 (113 upregulated genes and 25 downregulated genes in the tumor tissue; DEG4; Supplementary Table 3). These genes were then subjected to GO analysis. Upregulated DEG4 genes were enriched in GO terms related to extracellular matrix (ECM), collagen fibril organization and osteogenesis (Fig. 3a). The cancer-driving genes^5^ COL1A1, COL3A1, FAT4, and FKBP9, were commonly included in the DEGs with enriched GO terms. Although the number of downregulated DEG4 genes was small, GO analysis was performed. GO term of alpha-amino acid catabolic process and cellular amino acid catabolic process were enriched in the down regulated genes (Fig. 3b).

In DEG3 (268 genes in total), there were lots of gene (130 genes) that were not converted to human genes by RBH. Of these, 79 genes remain unannotated in *Xenopus* genome database. We then examined their orthologs just by best blast hits (the gene that came up first by the BLAST search), not by “reciprocal” best blast hit (RBH). Out of 130 genes, 73 genes were identified. The remaining 57 genes were putative homologs of the 73 genes or did not have any hits with the BLAST search. GO analysis was performed with the 73 genes in addition to the 138 genes (DEG4). The results in upregulated genes showed that the top enriched GO terms were almost consistent with the analysis conducted using the 138 genes alone (Fig. S2b,c).

### Analysis of differential gene expression of tumor tissue (flank skin) and the equivalent regions in no-tumor-bearing individuals

**Figure 4:**
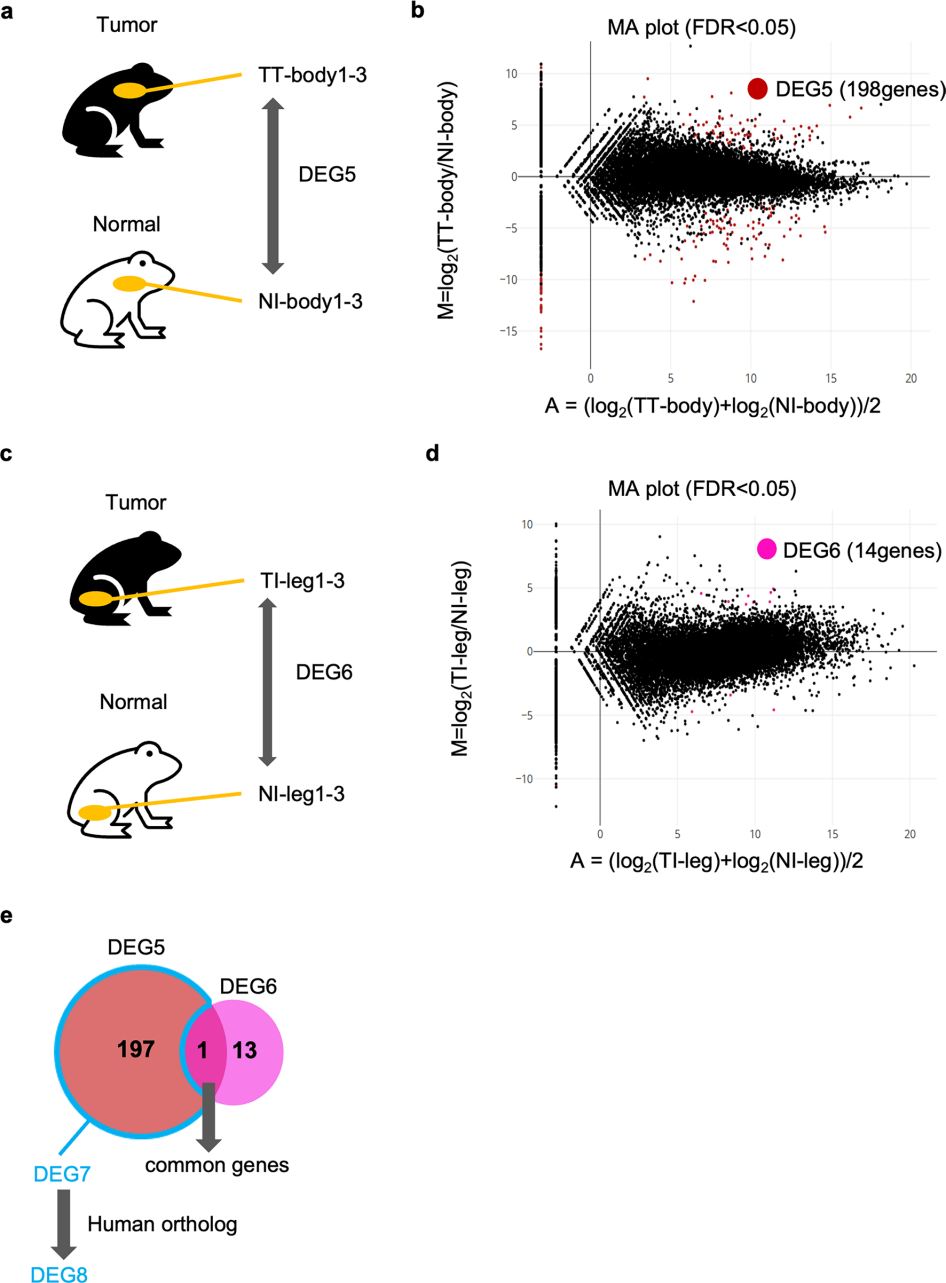
DEGs of TT-body1-3 vs NI-body1-3. (**a,b**) Schematic diagram of DEGs extraction (TT-body1-3 vs NI-body1-3) (**a**) and the MA plot (**b**). (**c,d**) Schematic diagram of DEGs extraction (TI-leg1-3 vs NI-leg1-3) (**c**) and the MA plot (**d**). Red (**b**) and pink (**d**) dots indicate DEGs with statistical significance (FDR < 0.05). (**e**) Venn diagram showing DEG7. DEG7 is DEG5—DEG5 ∩ DEG6.

As mentioned above, because the negative control leg skin was derived from tumor-bearing individuals, gene expression may differ from that in no-tumor-bearing individuals, even though the leg skin did not have tumors. Therefore, we compared gene expression with NI-body1-3 as a negative control (Fig. 4a). 198 genes were identified as DEGs (DEG5; Fig. 4b, Supplementary Table 4). Because these genes might include gene expression differences that caused by innate mutation throughout the body of tumor-bearing individuals, we excluded DEGs of TI-leg1-3 and NI-leg1-3 (DEG6; Supplementary Table 5) from DEG5 (Fig. 4c) to identify tumor-specific genes. 14 genes were identified as DEG6 (Fig. 4d). 1 gene was common to both DEG5 and DEG6 (Supplementary Table 6). Thus, by excluding the 1 gene from DEG5, 197 genes were defined as DEG7 (Supplementary Table 7), which are tumor-specific genes (Fig. 4e).

**Figure 5:**
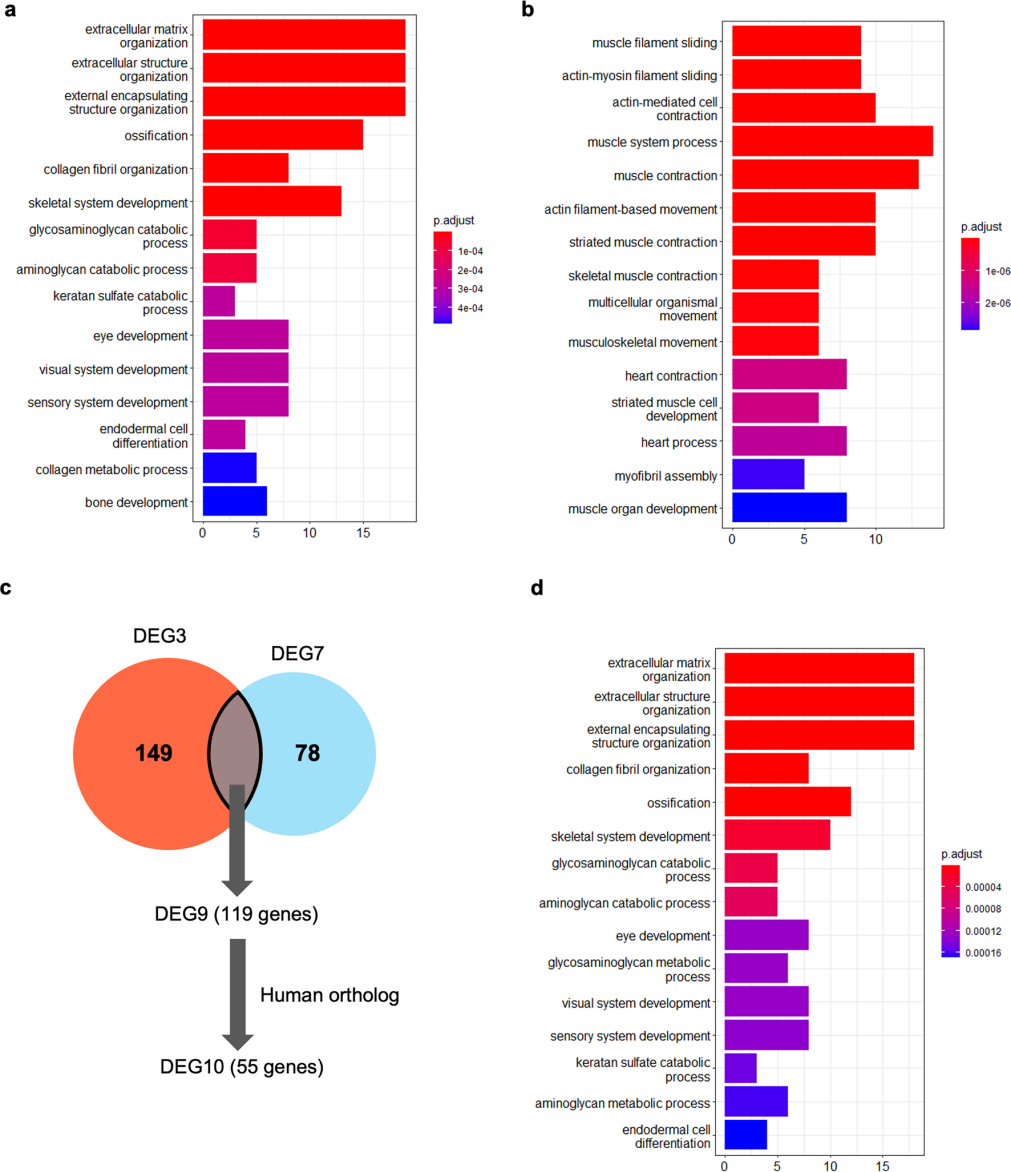
GO analysis of DEG8 and DEG10 genes. GO analysis of upregulated genes (**a**) or downregulated genes (**b**) of DEG8. Bar plots represents number of genes involved in each term. Adjusted p-value was represented by color scale, and the statistically significance level decreased from red (higher significance) to blue (lower significance). (**c**) Venn diagram showing DEG9, which is common genes of DEG3 and DEG7. (**d**) GO analysis of upregulated genes of DEG10.

We determined human orthologs of DEG7 by RBH, and 96 genes were identified (DEG8; Supplementary Table 8). Among the DEG8, 58 and 38 genes were upregulated and downregulated in tumor tissues, respectively. Upregulated DEG8 genes were enriched in GO terms related to the ECM, collagen metabolism, and bone formation, and these results were similar to those for DEG4 (Fig. 5a). In addition, the cancer-driving genes^5^ COL3A1and FAT4 were commonly included in DEG8 with enriched GO terms, similar to those of DEG4. Downregulated DEG8 genes were enriched in GO terms related to myogenic processes (Fig. 5b).

Non-tumor tissues (leg skin) of tumor-bearing individuals are possibly in a pre-tumor state. To examine the possibility, we extracted common genes between DEG5 and DEG6 and found only one gene, BAAT. To further examine it, we extracted common genes with gaining FDR threshold (FDR < 0.1). 22 genes were extracted: of them, 7 genes were reported to have roles in promoting or suppressing cancer in humans. For instance, AMPD3 is known to be highly expressed in gastrointestinal stromal tumors^19^; BGLAP is expressed in pancreatic cancer cells and promotes their growth and invasion^20^; COL6A6 inhibits the growth of non-small cell lung cancer^21^; PODN inhibits the growth of osteosarcoma^22^; and SOD3 is downregulated in breast cancer^23^.

To further narrow down responsible genes of the tumor in *Xenopus*, we investigated the genes commonly included in DEG3 and DEG7, which used different negative controls (Fig. 5c). Of these 119 genes (DEG9; Supplementary Table 9), 62 genes were upregulated, and 57 genes were downregulated in the tumor tissue. 55 genes were identified as human orthologs by RBH (DEG10; Supplementary Table 10), of which 46 genes were upregulated and 9 genes were downregulated. GO terms related to the ECM, collagen fibril organization and ossification were enriched in the upregulated genes (Fig. 5d). In the downregulated genes, GO term could not be properly extracted because of the small number of genes.

### Comparison of DEGs between *Xenopus* tumor tissues and human mesoderm-derived cancer tissues

We compared these expression patterns with those of human mesoderm-derived cancers because the tumor in *Xenopus* shows dermal expansion. Using human cancer expression data registered in The Cancer Genome Atlas (TCGA), we performed hierarchical clustering with all the expression data of TT-body1-3. TT-body1-3 seems to exhibit a similar pattern to human cancer tissues derived from bone, joint, and articular cartilage of the extremities (hereafter referred to as "cancer tissues derived from bone tissue"), connective tissue, subcutaneous tissue, and other soft tissues (Fig. S3a).

**Figure 6:**
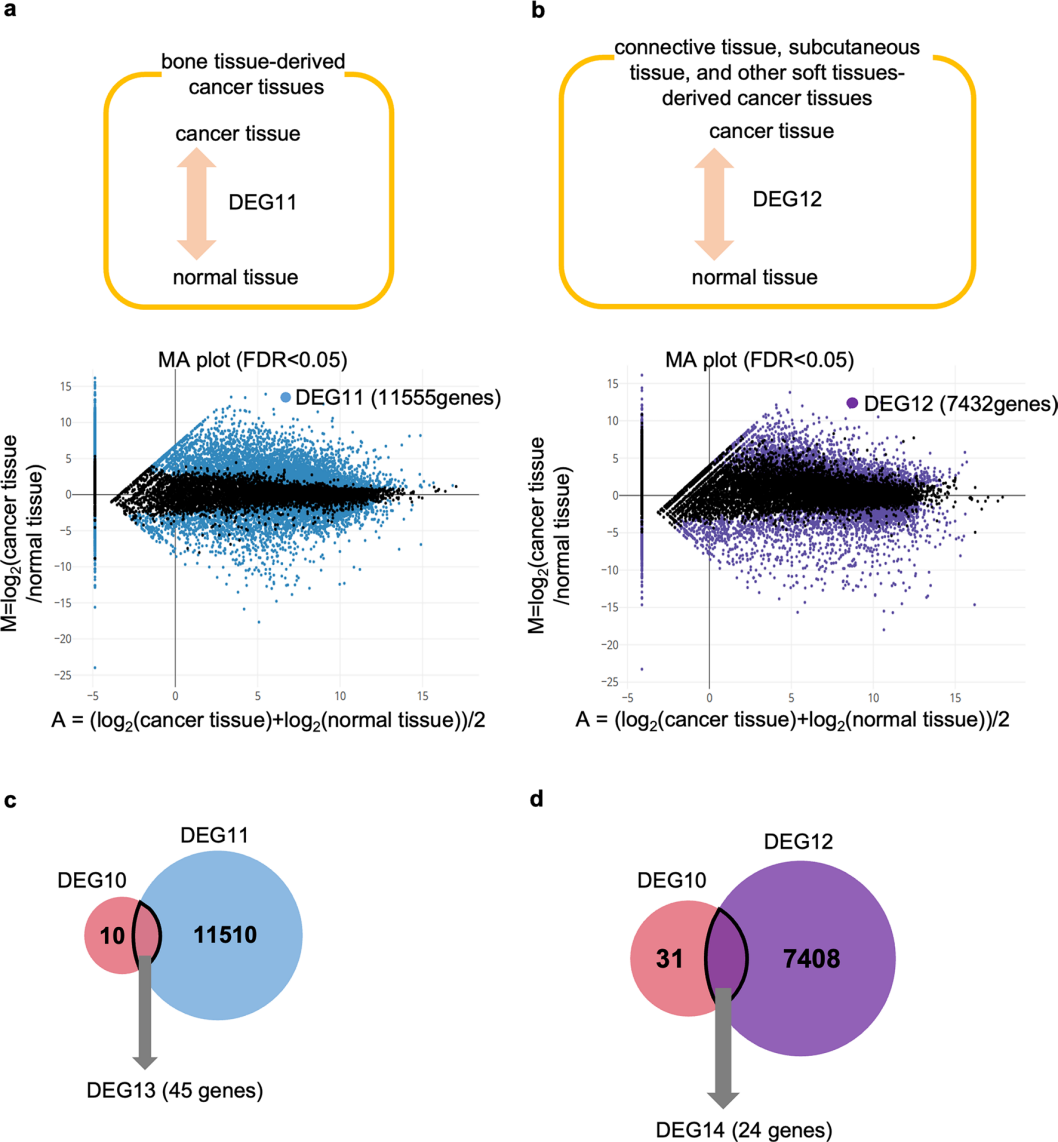
MA plot comparing gene expression pattern of cancer tissues with that of normal tissues in human. (**a,b**) MA plot of cancer tissue vs normal tissue derived from bone tissue (**a**) and connective tissues, subcutaneous tissue, and other soft tissues (**b**) in human. Blue (**a**) and purple (**b**) dots indicate DEGs with statistical significance (FDR < 0.05). (**c,d**) Venn diagram of the extraction of DEG13 (**c**) and DEG14 (**d**).

**Figure 7:**
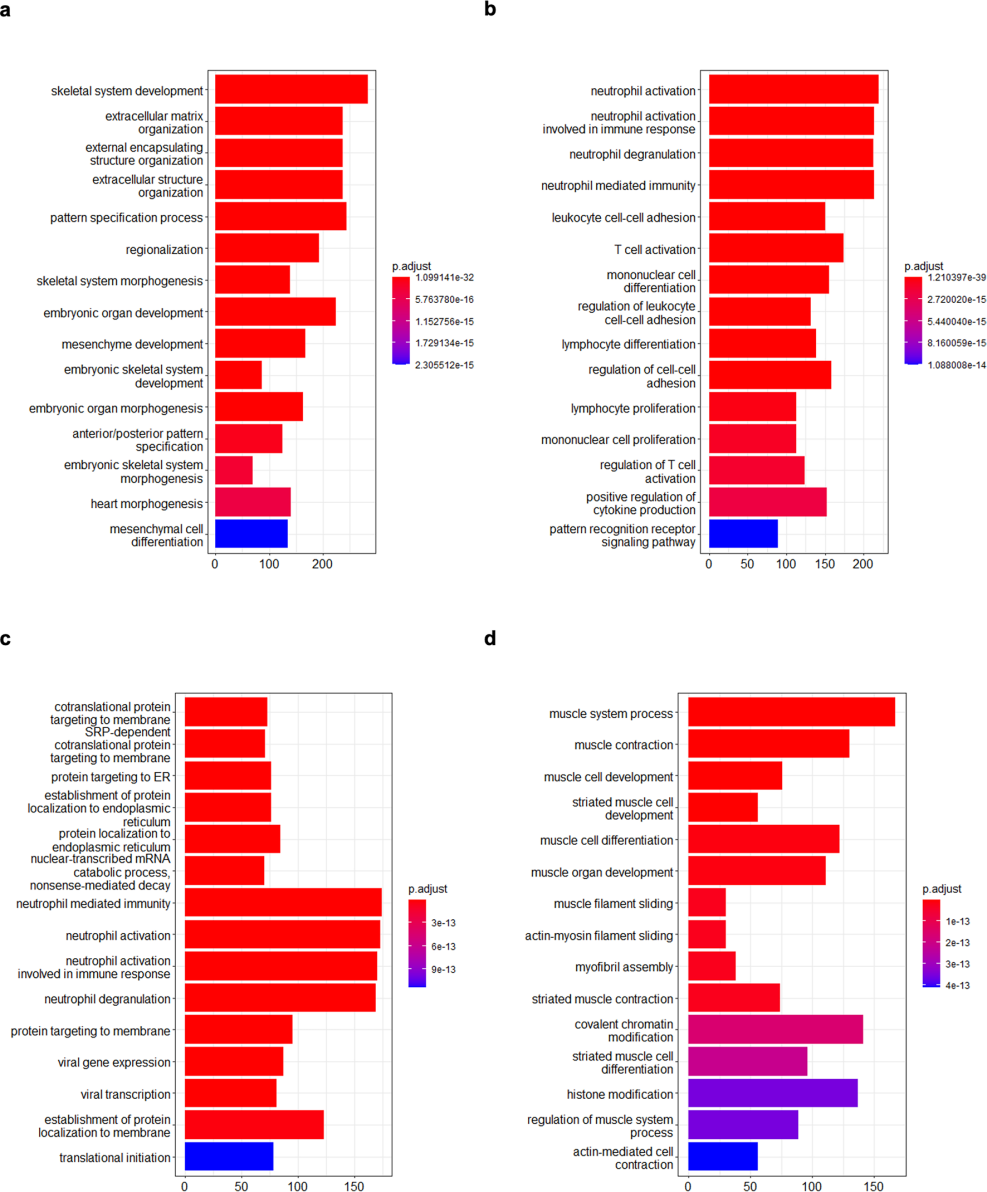
GO analysis of DEG11 and DEG12 genes. (**a,b**) GO analysis of upregulated genes (**a**) or downregulated genes (**b**) of DEG11. (**c,d**) GO analysis of upregulated genes (**a**) or downregulated genes (**b**) of DEG12. Bar plots represents number of genes involved in each term. Adjusted p-value was represented by color scale, and the statistically significance level decreased from red (higher significance) to blue (lower significance).

To compare the DEGs in *Xenopus* tumor tissues with those in human mesoderm-derived cancer, we identified DEGs in human cancer. Read-count data of bone marrow cells and muscle tissues were obtained as controls from the atlas of RNA-seq profiles for normal human tissues^24^. Consequently, 11,555 genes (7484 upregulated and 4071 downregulated genes) were identified as DEGs in the cancer tissues derived from bone tissue (DEG11, Fig. 6a, Supplementary Table 11), and 7432 genes (3787 upregulated and 3645 downregulated genes) were identified as DEGs in cancer tissues derived from connective, subcutaneous, and other soft tissues (DEG12, Fig. 6b, Supplementary Table 12). Similar to the *Xenopus* tumor, upregulated genes of DEG11 were enriched in GO terms related to ECM and skeletal system (Fig. 7a). Downregulated genes were enriched in GO terms related to T-cell activation and cell–cell adhesion (Fig. 7b). Upregulated genes of DEG12 were enriched in GO terms related to protein targeting to membrane (Fig. 7c). Downregulated genes were enriched in GO terms related to muscle tissue, such as the development of muscle structures and muscular system processes, as in the *Xenopus* tumor tissue (Figs. 5b, 7d).

We then identified common genes between *Xenopus* DEGs (DEG10) and human DEGs (DEG11) (Fig. 6c), which was 45 genes (DEG13; Supplementary Table 13). Of these genes, 41 were upregulated and 4 were downregulated in *Xenopus* tumor tissues. The cancer-driving genes^5^, COL3A1 and FAT4, were included in upregulated genes. We examined whether the expression of 45 genes was higher or lower in *Xenopus* tumor and human cancer than in each control. Most genes (44) showed similar expression patterns, upregulated or downregulated, in *Xenopus* and human tumor tissues (Fig. S3b,c). The remaining one gene, SOD3 was downregulated in *Xenopus* tumor whereas it was upregulated in human cancer.

We then extracted common DEGs from DEG10 and DEG12 and identified 24 common genes (DEG14; Supplementary Table 14; Fig. 6d), which included 22 upregulated and 2 downregulated genes in *Xenopus* tumor tissue. Similar to DEG13, DEG14 included the cancer-driving genes^5^ COL3A1 and FAT4. In addition, 19 of the 22 genes showed similar gene expression patterns, upregulated or downregulated, in *Xenopus* tumor and human cancer derived from connective, subcutaneous, and other soft tissues (Fig. S3d,e). About the remaining genes, FAT4 was upregulated in *Xenopus* tumor and downregulated in human cancer. Two genes, TFF3 and FCGBP, were downregulated in *Xenopus* tumor and upregulated in human cancer.

### Identification of DEGs in each *Xenopus* tumor tissue

Although the three samples derived from the NH strain in *Xenopus* were considered biological replicates in the above analyses, each tumor tissue may be in a different tumor progression state. At least, based on the external view, TT-body3 (NH-V-1) may be different from the other tumors: TT-body3 exhibited a spherical tumor (Fig. 1b’), while TT-body1 (NH-VIII-7) and TT-body2 (NH-III-4) exhibited distorted shapes with multiple overlapping protrusions (Fig. 1a’, Fig. S4a).

To investigate this, we identified DEGs of each TT-body with the same negative control samples (leg skin of tumor-bearing individuals [TI-leg1-3] or flank skin of no-tumor-bearing individuals [NI-body1-3]; Fig. S4b–g, Supplementary Tables 15–18). The number of DEGs in TT-body3 was higher than in TT-body #1 (TT-body1) or TT-body2, especially the DEGs in tumor tissue vs. leg skin (Fig. S4b,d,f). To further determine the differences among these DEGs, we extracted the common genes of each DEGs identified in the leg skin as controls (Fig. S5a,b). The percentage of common DEGs between TT-body1 and TT-body2 was larger than 50% (Fig. S5a, bars 1–2). The percentage of common DEGs from TT-body1 and 3 was 65% of that from TT-body1 (Fig. S5a, bar 3), and 62% of the DEGs from TT-body2 were common to DEGs from TT-body2 and TT-body3 (Fig. S5a, bars 5). These results suggest that DEGs from TT-body1 and TT-body2 share many common genes and that DEGs of TT-body3 vs. TI-leg1-3 also share genes with TT-body1 and TT-body2.

The number of DEGs in TT-body3 was higher than that in TT-body1 and TT-body2, although there were many common genes among the DEGs in TT-body1 to TT-body3, suggesting that TT-body3 is in a higher state of tumor progression than TT-body1 and TT-body2. To examine TT-body3 specific genes, 3903 genes not included in DEGs of TT-body1 and TT-body2 were extracted and converted to human genes (2575 genes). In the 2575 genes, it was found that 123 genes were cancer-driving genes^5^.

### Pathway analysis of DEG in *Xenopus*

**Figure 8:**
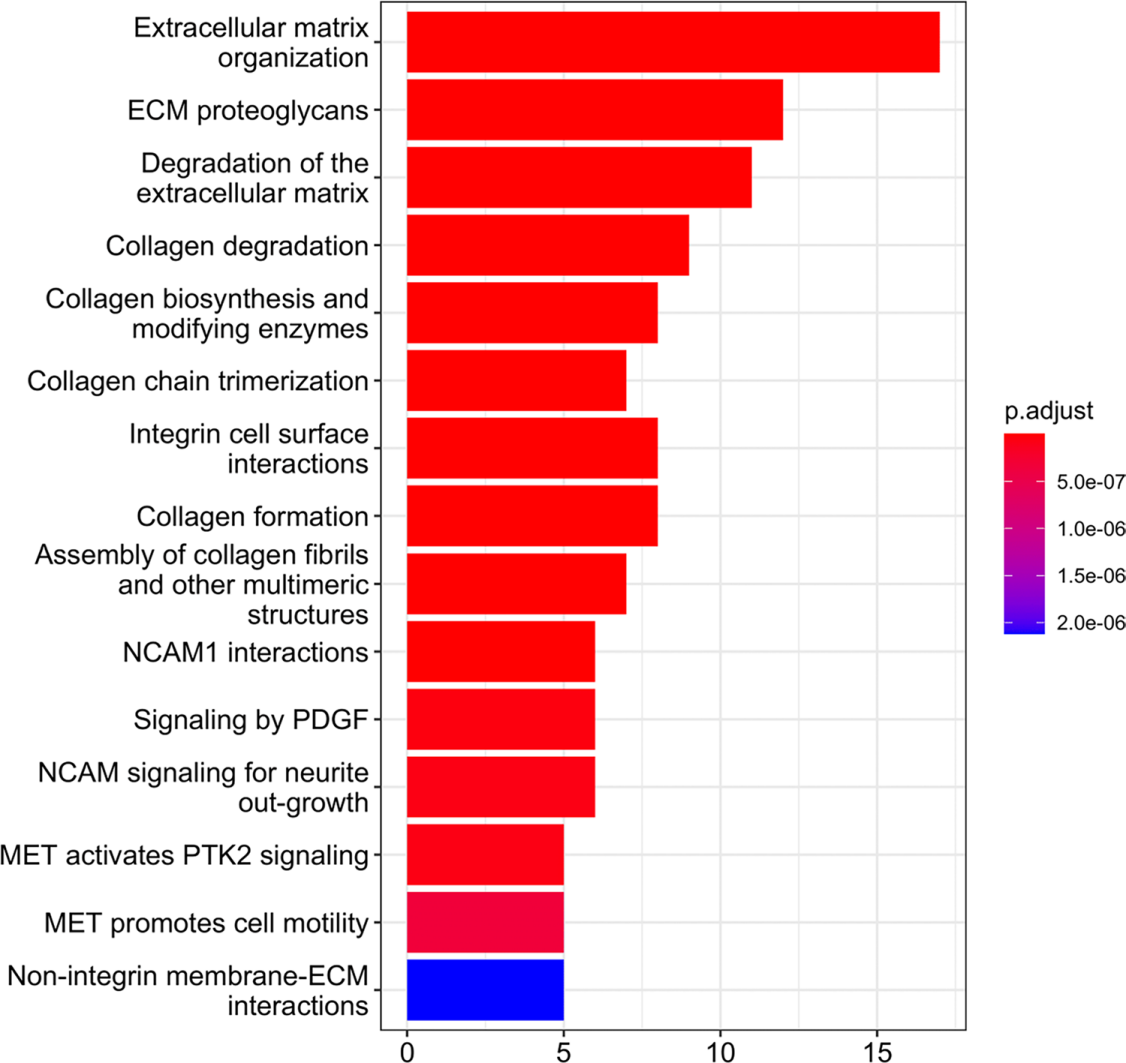
Reactome Pathway Analysis of genes of DEG10. Bar plots represents number of DEGs involved in each term. Adjusted p-value was represented by color scale, and the statistically significance level decreased from red (higher significance) to blue (lower significance).

As described above, we found that the tumor-specific genes in *Xenopus*, DEG10 (common genes between DEG4 (tumor tissue vs. leg skin) and DEG8 (tumor tissue vs. flank skin)), shared many genes with the cancer-specific genes in humans (DEG11 and 12). To further investigate DEG10, we performed pathway analysis of DEG10 using ReactomePA^25^, a database of human biological reactions and pathways. Genes involved in ECM and collagen fibers were enriched (Fig. 8). PDGF and MET signaling factors related to angiogenesis in tumor tissues were also enriched. As genes participating in multiple pathways are probably effective in tumorigenesis, we determined the number of genes participating in multiple pathways (> 2 pathways) in DEG10 (Fig. S6a,b). Of the 55 genes (DEG10), 19 genes, most of which were upregulated in *Xenopus* tumor tissue, were involved in multiple pathways (Fig. S6a). For instance, COL1A2 and COL3A1 function in many pathways such as ECM organization, collagen fibers, MET signaling, and PDGF signaling. LUM is involved in multiple functions such as collagen fiber organization, epithelial cell migration, and tissue repair and exhibits dual functionality as both an oncogene and a tumor suppressor gene^26,27^.

To confirm the involvement of collagen in the tumors, we performed Elastica van Gieson staining with *Xenopus* tumors that have similar characteristics (Fig. S7a–c). This was clearly stained, indicating the presence of collagen fiber hyperplasia in the tumor (Fig. S7d). The tumor tissue in the dermis was also clearly stained by Azan staining (Fig. S7e), indicating that the collagen fibers in the tumor include type I collagen. The dermis was also stained by silver, indicating that this collagen fibers also include type III collagen (Fig. S7f). These characteristics are similar to dermatofibromas in human, which sometimes metastasizes^28^.

### Survival curves of human patients with defects in the expression of DEG10 genes

Among all the genes of DEG10 (55 genes), 9 genes have not been well analyzed in the cancer field. We analyzed the survival curves of human patients with defects in the expression levels of these genes using Survival Genie^29^ (Fig. S8, Supplementary Table 19), which uses human expression data from many cancer tissues obtained from The Cancer Genome Atlas (TCGA). For example, in patients with kidney cancer, higher expression of LACTBL1 was correlated with a decrease in survival rates (Fig. S8d). Consistent with this, LACTBL1 was highly expressed in *Xenopus* tumors. In patients with colon, connective, subcutaneous, and other soft tissue cancers, higher expression of KERA, GUSB, and LACTBL1 had decreased survival rates (Fig. S8i), which were consistently highly expressed in *Xenopus* tumors. In patients with cancer of the bones, joints, the articular cartilage of limbs, lower expression of KLHDC7A, whose expression was also lower in *Xenopus* tumors, had decreased survival rates (Fig. S8h). Taken together, 6 out of the 9 genes were proposed to influence the survival rate in human cancer.

## Discussion

In this study, we performed histological and RNA-seq analyses on tumor tissues in *Xenopus*. We have identified putative responsible genes for tumorigenesis, some of which correlate with prognosis in human cancer patients.

In the historical analyses, dermal region seemed to have increased in the tumor tissues (Fig. 1d,e). Considering the dermis consists of various cell types, it is important to determine which part was increased. In the GO analyses of upregulated genes in tumor tissues, the terms of ossification and collagen fibril organization were enriched. The layer below the granules in the dermis, the Eberth–Katschenko layer, is known for its high levels of calcium^30^ and collagen^31,32^, and the surrounding region of the layer is also suggested to be high in the expression of osteogenic genes^30^. Taken together, it is plausible that these regions have expanded in the tumor tissues. To elucidate the underlying mechanism of this expansion, two potential reasons can be considered: promoted proliferation or enhanced/changed differentiation. Taking the absence of proliferation-related terms in the GO analysis into consideration, the latter has higher possibility.

Although most cancer research focuses on gene mutations, alterations in gene expression, must have a substantial impact on tumorigenesis. By focusing on gene expression of tumors, we have successfully identified novel putative responsible genes (DEG10) involved in cancer formation. In addition, most genes (46 genes) in DEG10 (55 genes) related to *Xenopus* tumorigenesis are involved in human oncogenesis, suggesting that there are high similarities between *Xenopus* and humans in the mechanism of tumorigenesis, and *Xenopus* could be a model organism for human cancer research. In addition, we found that the expression levels of 6 genes in DEG10, which have not been well analyzed in relation to human cancer, impacted the subsequent survival rate of cancer patients in human (Fig. S8). The reason why these genes have not been identified as cancer-driving genes^5^ to date may be partly attributed to their infrequent occurrence of mutations in human cancers or mutation-based analyses primarily focus on the coding sequences, not on the expression levels. Therefore, a drug capable of regulating the expression level of these genes could potentially control cancer progression. This could be a novel option for drug selection, which based on the gene expression in the tumor. Thus, this study provides a new perspective in the field of human cancer treatment.

## Materials and methods

### *Xenopus tropicalis *(*X. tropicalis*)

All animal experiments were approved by the Office for Life Science Research Ethics and Safety, at the University of Tokyo (#2020-6). Tumor and normal individuals of *X. tropicalis* NH (Nigerian H) strain, a fully inbred strain^33^, were provided by the Amphibian Research Center (Hiroshima University, Japan). Detailed information on the analyzed individuals is shown in Supplementary Table 20. In this study, we analyzed only females of *Xenopus*.

### Imaging, histology

Tissues were fixed at room temperature overnight using Bouin's Fixative. The fixed tissues were then embedded in paraffin, and 8 µm sections were generated by microtomy. Standard methods were used to stain the sections with hematoxylin and eosin (HE). Collagen was stained with Elastica van Gieson (EvG) staining. Type I collagen fibers were stained blue with Azan staining. Type III collagen was stained black with silver staining.

### RNA purification

Tumor tissues and skin of the leg and flank were surgically collected and subsequently crushed using a bead crusher (MS-100R, TOMY) with 6 mm diameter beads. The beads and samples were placed in a tube together and crushed five times (3000 rpm, 1 min, 2 °C). RNA was then purified using the ReliaPrep RNA Cell Miniprep System (Promega).

### RNA sequencing

Sequencing was performed with Illumina NovaSeq6000. Sequences were aligned by HISAT2^34^. Samtools^35^ was used to convert SAM files to indexed, sorted, and merged BAM files. Transcripts were assembled and quantified using StringTie^36^.

### Hierarchical clustering

To compare gene expression patterns between samples, hierarchical clustering with Pearson’s correlation coefficient^15^ was performed using R software. R function ‘hclust’ was used for sample clustering based on gene expression matrices. The distance matrix is based on 1 − r, where r is the Pearson’s correlation coefficient between sample pairs. Ward’s minimum variance method was used as the agglomeration method. The Ward method is used to calculate the distance matrix between clusters, and the length of each branch of the dendrogram is represented by "Height".

### DEG output and MA plot

The read counts were normalized using the trimmed mean of M values (TMM) method^16^ with the edgeR Bioconductor package. This method scales read counts by the weighted log fold-change values of a reference sample with genes that have extreme log-fold-changes (M values) and extreme absolute expression levels (A values) removed from data. Differentially expressed genes (DEGs) were identified using a false discovery rate (FDR < 0.05). The TCC^37,38^ package was used to calculate M and A values for the MA plot. TCC is an R package that provides functions for differential expression analysis of tag count data. We used the TCC-GUI^39^, a graphical user interface for TCC, to analyze the data.

### OrthoFinder

Orthofinder is an algorithm for inferring orthologs across multiple species^17,18^. FASTA files of protein sequence in *Xenopus* and human were downloaded from the NCBI Reference Sequence Database (RefSeq), and the sequences were used as the query for the followings. Reciprocal best BLAST Hits (RBH) method was applied to identify human orthologs for each gene in *Xenopus*.

### RNA-seq data from human cancer patients

RNA-seq read count information for 43 different tumor types, which are all the categories of cancer available in the database, were collected from Genomic Data Commons (GDC) Data Portal of the Cancer Genome Atlas Program (TCGA) (10 data were obtained from each cancer data collection). We used TCGA’s "Open Access data," which does not require user certification. These read count data were normalized by the TMM method^16^.

### Gene Ontology analysis and pathway analysis

To perform Gene Ontology (GO) analysis, we referred to GO-term annotation for human genome. We examined human orthologs of the *Xenopus* gene and Gene Ontology enrichment analysis was performed using clusterprofiler^40^, a package implemented in R. Reactome pathway analysis was performed using ReactomePA, an R package based on the human REACTOME pathway database^25^.

### Survival rate analysis

We utilized Survival Genie^29^, a web-based tool, to perform Kaplan–Meier analysis, which is a statistical method to evaluate survival rates, on single-cell RNA-seq (scRNA-seq) data and a variety of other molecular inputs such as gene sets, genes ratio, tumor infiltrating immune cells proportion, gene expression profile scores, and tumor mutation burden, based on cancer patient data derived from 33 projects of TCGA’s Open Access data. We defined high/low expression groups using “median” among selected samples. We also defined as a significant difference when it has > 0.2 difference in survival probability between high and low expression of a certain gene.

### Supplementary Information

Supplementary Figures.

Supplementary Table 1.

Supplementary Table 2.

Supplementary Table 3.

Supplementary Table 4.

Supplementary Table 5.

Supplementary Table 6.

Supplementary Table 7.

Supplementary Table 8.

Supplementary Table 9.

Supplementary Table 10.

Supplementary Table 11.

Supplementary Table 12.

Supplementary Table 13.

Supplementary Table 14.

Supplementary Table 15.

Supplementary Table 16.

Supplementary Table 17.

Supplementary Table 18.

Supplementary Table 19.

Supplementary Table 20.

## Data Availability

The raw RNA-seq datasets of *Xenopus* tissue (fastq format) have been deposited to NCBI-GEO with the accession number GSE233287 (https://www.ncbi.nlm.nih.gov/geo/query/acc.cgi?acc=GSE233287).

## References

[1] Palladino-Davis AG, Mendez BM, Fisichella PM, Davis CS (2015). Dietary habits and esophageal cancer. Dis. Esophagus.

[2] Gandini S (2008). Tobacco smoking and cancer: A meta-analysis. Int. J. Cancer.

[3] Rumgay H, Murphy N, Ferrari P, Soerjomataram I (2021). Alcohol and cancer: Epidemiology and biological mechanisms. Nutrients.

[4] Bozic I (2010). Accumulation of driver and passenger mutations during tumor progression. Proc. Natl. Acad. Sci. U. S. A..

[5] Sondka Z (2018). The COSMIC Cancer Gene Census: Describing genetic dysfunction across all human cancers. Nat. Rev. Cancer.

[6] Ford D (1998). Genetic heterogeneity and penetrance analysis of the BRCA1 and BRCA2 genes in breast cancer families. The Breast Cancer Linkage Consortium. Am. J. Hum. Genet..

[7] Fearon ER, Vogelstein B (1990). A genetic model for colorectal tumorigenesis. Cell.

[8] Hanahan D, Weinberg RA (2011). Hallmarks of cancer: The next generation. Cell.

[9] Zhou Q (2021). Genes that predict poor prognosis in breast cancer via bioinformatical analysis. Biomed. Res. Int..

[10] Zhang J, Liu D, Li L, Wang Q, Lan Y (2020). Detection of critical genes associated with poor prognosis in breast cancer via integrated bioinformatics analyses. JBUON.

[11] Jinek M (2012). A programmable dual-RNA-guided DNA endonuclease in adaptive bacterial immunity. Science.

[12] Han K (2020). CRISPR screens in cancer spheroids identify 3D growth-specific vulnerabilities. Nature.

[13] Nakayama T (2014). Cas9-based genome editing in *Xenopus tropicalis*. Methods Enzymol..

[14] Suzuki, M., Igawa, T., Suzuki, N., Ogino, H. & Ochi, H. Spontaneous neoplasia in the western clawed frog Xenopus tropicalis. *MicroPubl Biol* **2020**, (2020). 10.17912/micropub.biology.000294 PMC7474949 32908966

[15] Kim T (2019). Impact of similarity metrics on single-cell RNA-seq data clustering. Brief Bioinform..

[16] Maza E (2016). In Papyro comparison of TMM (edgeR), RLE (DESeq2), and MRN normalization methods for a simple two-conditions-without-replicates RNA-Seq experimental design. Front. Genet..

[17] Emms DM, Kelly S (2019). OrthoFinder: Phylogenetic orthology inference for comparative genomics. Genome Biol..

[18] Emms DM, Kelly S (2015). OrthoFinder: Solving fundamental biases in whole genome comparisons dramatically improves orthogroup inference accuracy. Genome Biol..

[19] Wong M (2017). AMPD3 is associated with the malignant characteristics of gastrointestinal stromal tumors. Oncol. Lett..

[20] Kayed H (2007). BGLAP is expressed in pancreatic cancer cells and increases their growth and invasion. Mol. Cancer.

[21] Qiao H, Feng Y, Tang H (2021). COL6A6 inhibits the proliferation and metastasis of non-small cell lung cancer through the JAK signalling pathway. Transl. Cancer Res..

[22] Han X (2022). Hypermethylated PODN represses the progression of osteosarcoma by inactivating the TGF-β/Smad2/3 pathway. Pathol. Res. Pract..

[23] Zhang Y (2022). The effect of extracellular superoxide dismutase (SOD3) gene in lung cancer. Front. Oncol..

[24] Suntsova M (2019). Atlas of RNA sequencing profiles for normal human tissues. Sci. Data.

[25] Yu G, He QY (2016). ReactomePA: An R/Bioconductor package for reactome pathway analysis and visualization. Mol. Biosyst..

[26] Chen X (2020). LUM expression and its prognostic significance in gastric cancer. Front. Oncol..

[27] Nikitovic D, Katonis P, Tsatsakis A, Karamanos NK, Tzanakakis GN (2008). Lumican, a small leucine-rich proteoglycan. IUBMB Life.

[28] Mentzel T (2013). Malignant dermatofibroma: Clinicopathological, immunohistochemical, and molecular analysis of seven cases. Mod. Pathol..

[29] Dwivedi B, Mumme H, Satpathy S, Bhasin SS, Bhasin M (2022). Survival Genie, a web platform for survival analysis across pediatric and adult cancers. Sci. Rep..

[30] Okada M, Tazawa I, Nakajima K, Yaoita Y (2013). Expression of the amelogenin gene in the skin of *Xenopus tropicalis*. Zoolog. Sci..

[31] Greven H, Zanger K, Schwinger G (1995). Mechanical properties of the skin of Xenopus laevis (Anura, Amphibia). J. Morphol..

[32] Taylor RE, Taylor HC, Barker SB (1966). Chemical and morphological studies on inorganic phosphate deposits in Rana catesbeiana skin. J. Exp. Zool..

[33] Igawa T (2015). Inbreeding ratio and genetic relationships among strains of the western clawed frog, *Xenopus tropicalis*. PLoS One.

[34] Kim D, Langmead B, Salzberg SL (2015). HISAT: A fast spliced aligner with low memory requirements. Nat. Methods.

[35] Li H (2009). The sequence Alignment/Map format and SAMtools. Bioinformatics.

[36] Pertea M (2015). StringTie enables improved reconstruction of a transcriptome from RNA-seq reads. Nat. Biotechnol..

[37] Sun J, Nishiyama T, Shimizu K, Kadota K (2013). TCC: An R package for comparing tag count data with robust normalization strategies. BMC Bioinform..

[38] Tang M, Sun J, Shimizu K, Kadota K (2015). Evaluation of methods for differential expression analysis on multi-group RNA-seq count data. BMC Bioinform..

[39] Su W, Sun J, Shimizu K, Kadota K (2019). TCC-GUI: A Shiny-based application for differential expression analysis of RNA-Seq count data. BMC Res. Notes.

[40] Yu G, Wang LG, Han Y, He QY (2012). ClusterProfiler: An R package for comparing biological themes among gene clusters. OMICS.

