# Supplementary material for: Identification of tumor-related genes via RNA sequencing of tumor tissues in Xenopus tropicalis
Source: Sci Rep. 2023 Aug 14;13:13214. doi: 10.1038/s41598-023-40193-7 (PMC10425369; doi:10.1038/s41598-023-40193-7)
Supplement: Supplementary file 1 — Supplementary Figures. [file 41598_2023_40193_MOESM1_ESM.pdf]

Figure S1

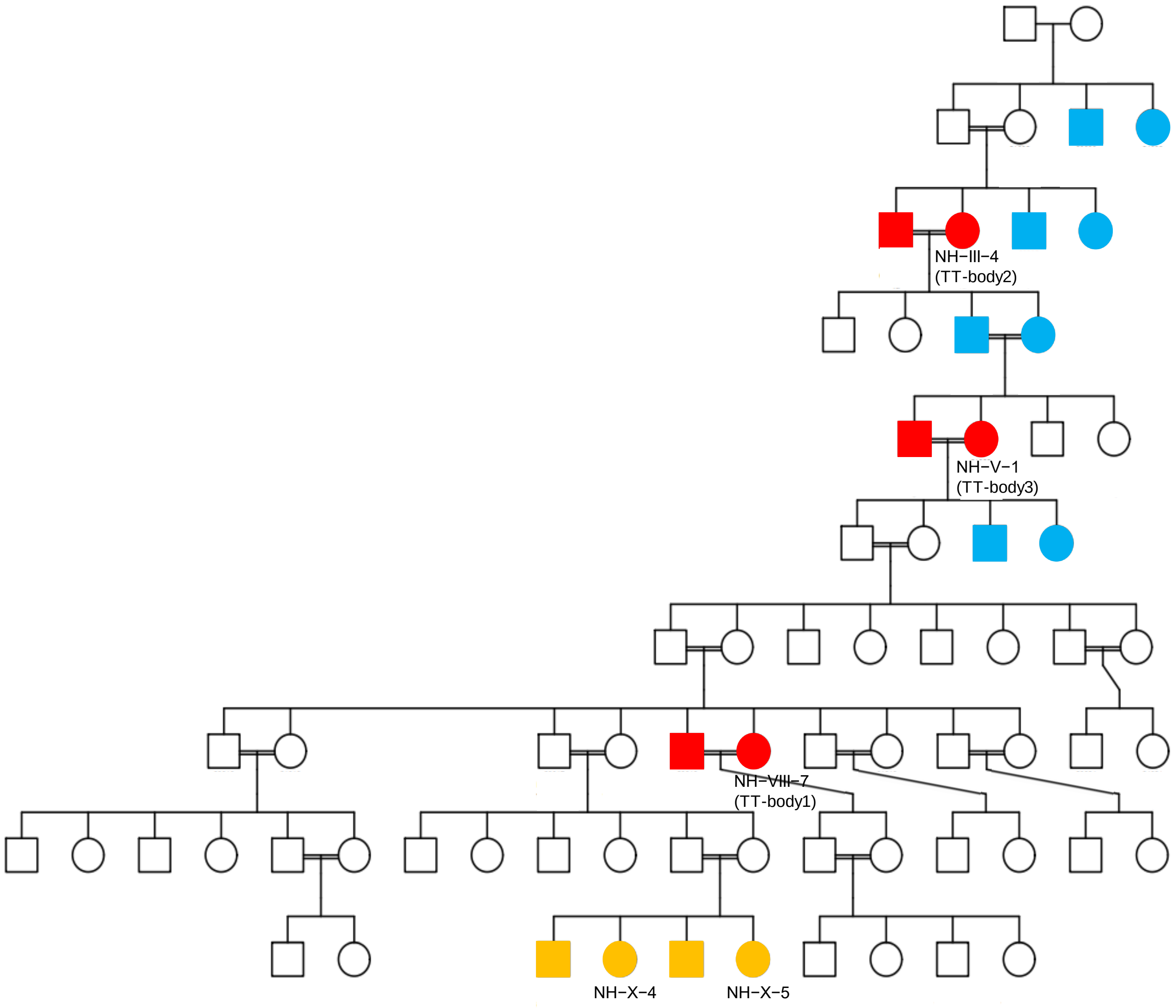

Fig. S1 Pedigree chart of NH strain in *X. tropicalis*.

Red color indicates the colonies in which tumor individuals were found and analyzed, while blue color indicates the colonies in which tumor individuals were found but were not analyzed. Orange color represents the colonies that has no-tumor-bearing individuals, serving as negative control samples. Circles denote female colonies, while squares denote male colonies. The colony names that we analyzed in this study, which are all female colonies, were indicated below the circles.

Figure S2

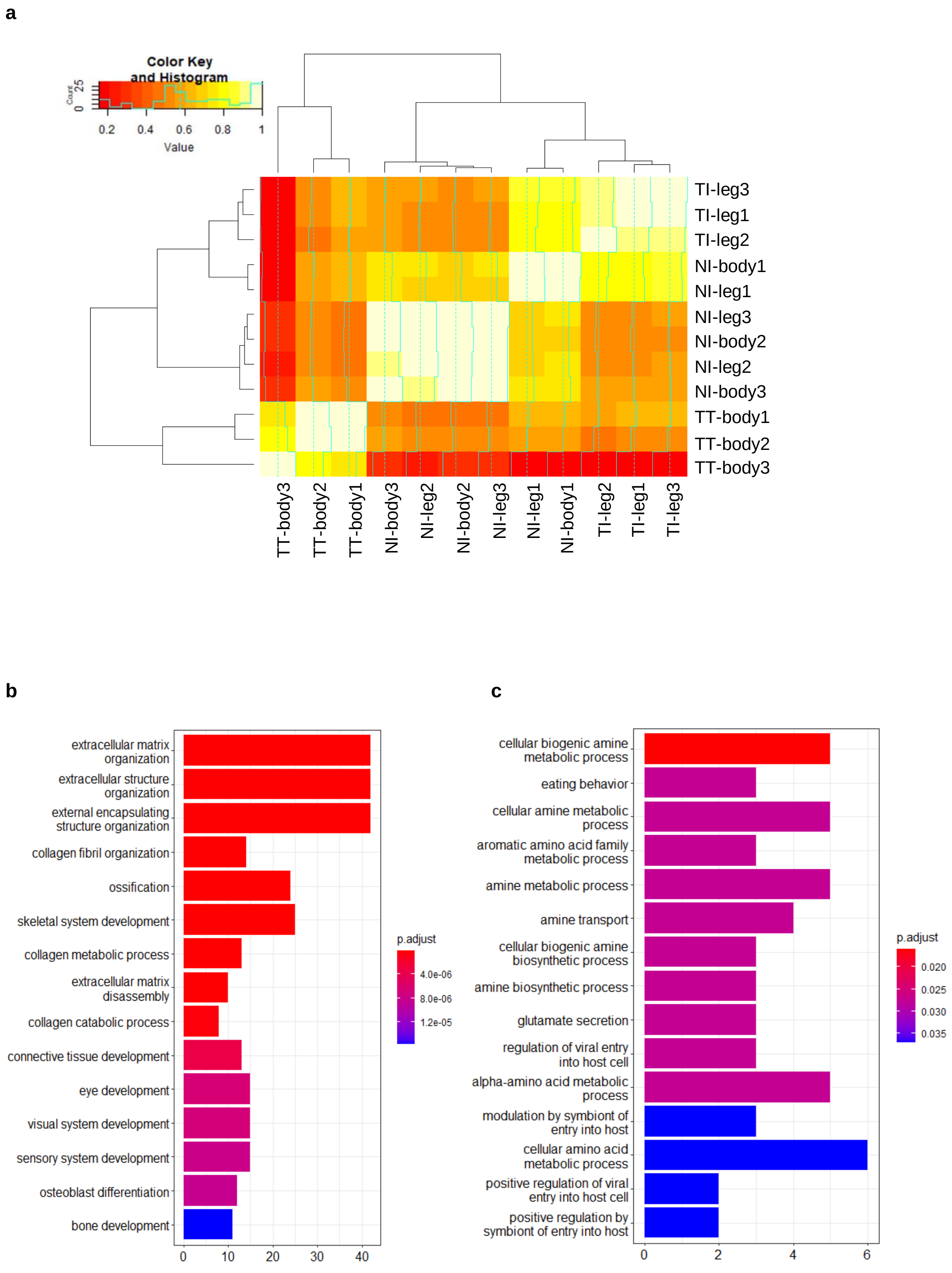
a
Color Key
and Histogram
Count
0
25
0.2
0.4
0.6
0.8
1
Value
TI-leg3
TI-leg1
TI-leg2
NI-body1
NI-leg1
NI-leg3
NI-body2
NI-leg2
NI-body3
TT-body1
TT-body2
TT-body3
TT-body3
TT-body2
TT-body1
NI-body3
NI-leg2
NI-body2
NI-leg3
NI-leg1
NI-body1
TI-leg2
TI-leg1
TI-leg3
b
extracellular matrix organization
extracellular structure organization
external encapsulating structure organization
collagen fibril organization
ossification
skeletal system development
collagen metabolic process
extracellular matrix disassembly
collagen catabolic process
connective tissue development
eye development
visual system development
sensory system development
osteoblast differentiation
bone development
0
10
20
30
40
p.adjust
4.0e-06
8.0e-06
1.2e-05
c
cellular biogenic amine metabolic process
eating behavior
cellular amine metabolic process
aromatic amino acid family metabolic process
amine metabolic process
amine transport
cellular biogenic amine biosynthetic process
amine biosynthetic process
glutamate secretion
regulation of viral entry into host cell
alpha-amino acid metabolic process
modulation by symbiont of entry into host
cellular amino acid metabolic process
positive regulation of viral entry into host cell
positive regulation by symbiont of entry into host
0
2
4
6
p.adjust
0.020
0.025
0.030
0.035

Fig. S2 Hierarchical clustering of all analyzed samples with heatmaps and GO analysis with genes of DEG4 that was extracted by best blast hits.

(a) Hierarchical clustering of analyzed samples with heatmaps. Darker color indicates a sample pair that has differential expression pattern (Pearson's correlation coefficient: white=1, red=0). (b-c) GO analysis with genes of DEG4 that was extracted by best blast hits (the gene that came up first by the BLAST search), not by "reciprocal" best blast hit (RBH) of DEG3 genes. Up-regulated genes (b) and down-regulated genes (c) in tumor tissues. Bar plots represents number of genes involved in each term. Adjusted p-value was represented by color scale, and the statistically significance level decreased from red (higher significance) to blue (lower significance).

Figure S3

a

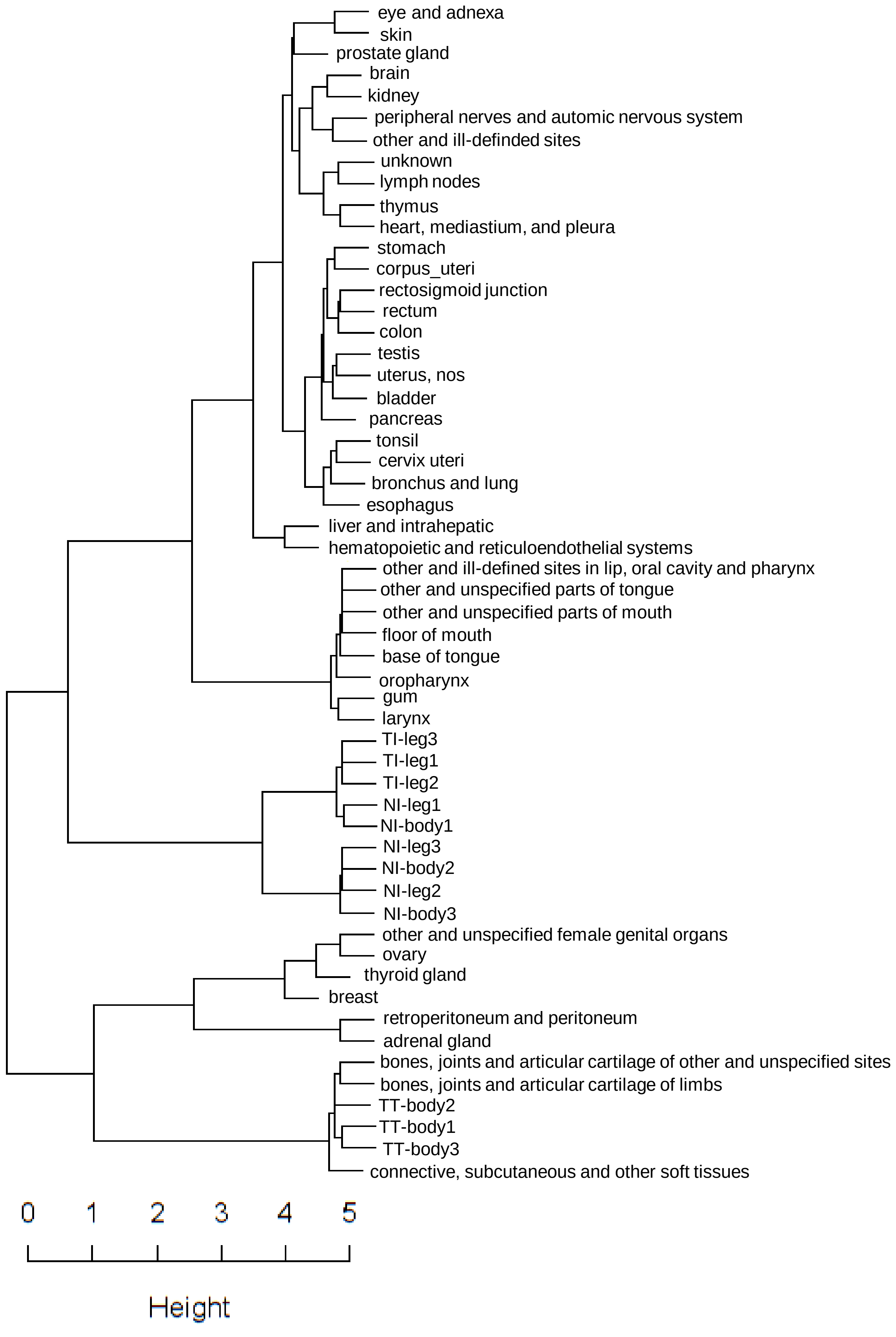
eye and adnexa
skin
prostate gland
brain
kidney
peripheral nerves and automic nervous system
other and ill-definded sites
unknown
lymph nodes
thymus
heart, mediastium, and pleura
stomach
corpus_uteri
rectosigmoid junction
rectum
colon
testis
uterus, nos
bladder
pancreas
tonsil
cervix uteri
bronchus and lung
esophagus
liver and intrahepatic
hematopoietic and reticuloendothelial systems
other and ill-defined sites in lip, oral cavity and pharynx
other and unspecified parts of tongue
other and unspecified parts of mouth
floor of mouth
base of tongue
oropharynx
gum
larynx
TI-leg3
TI-leg1
TI-leg2
NI-leg1
NI-body1
NI-leg3
NI-body2
NI-leg2
NI-body3
other and unspecified female genital organs
ovary
thyroid gland
breast
retroperitoneum and peritoneum
adrenal gland
bones, joints and articular cartilage of other and unspecified sites
bones, joints and articular cartilage of limbs
TT-body2
TT-body1
TT-body3
connective, subcutaneous and other soft tissues
0
1
2
3
4
5
Height

Figure S3

b

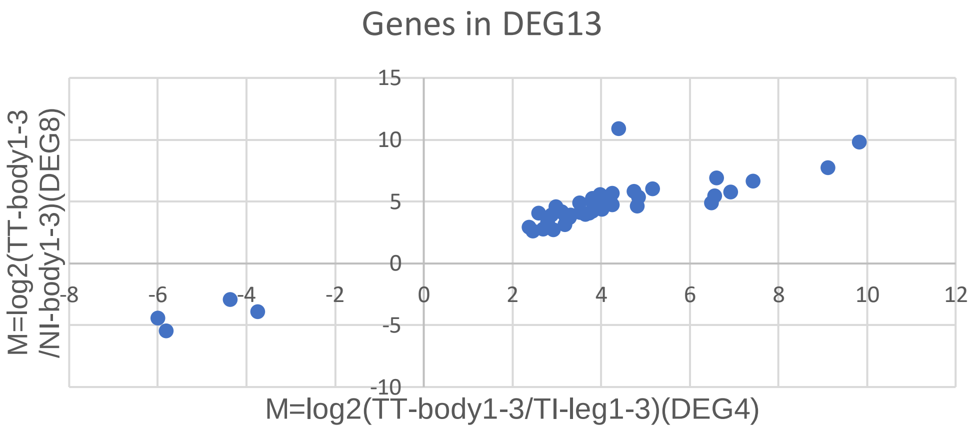

c

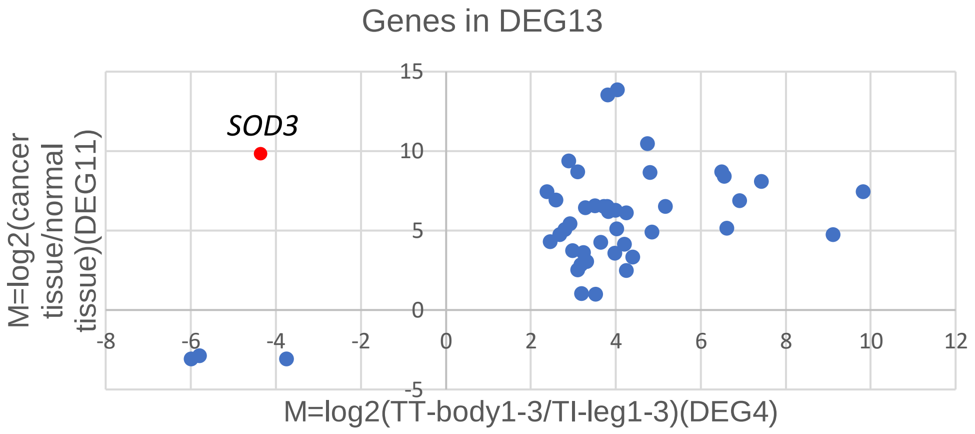

d

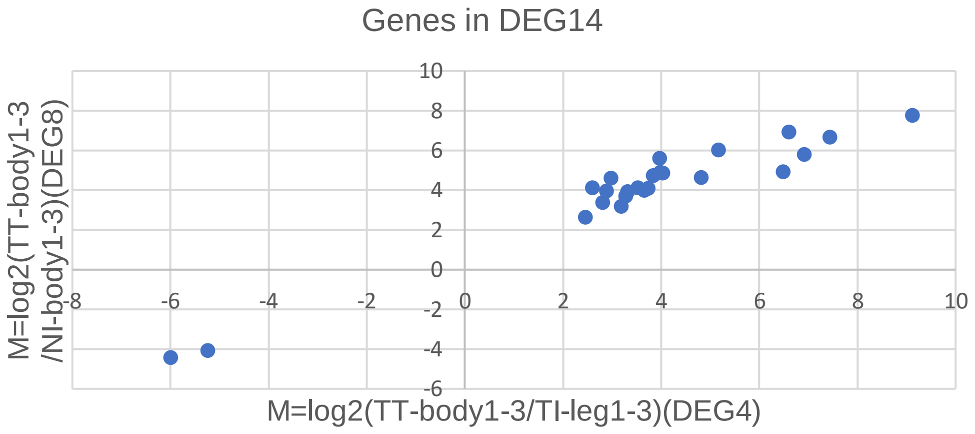

e

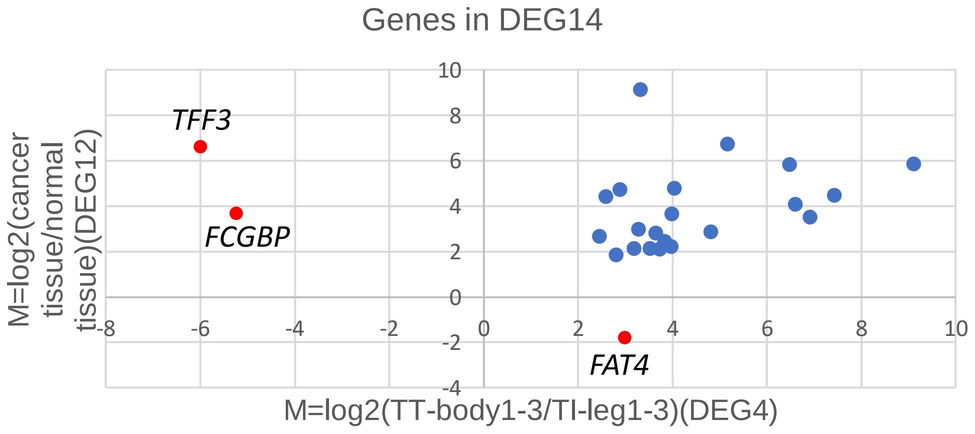

Fig. S3 Differences in gene expression patterns of TT-body1 to 3 and human cancer tissues.

(a) Hierarchical clustering of all analyzed tissues of *Xenopus* and 43 different human cancer tissues obtained from TCGA database, using Pearson's correlation coefficient. (b) Expression pattern of DEG13 genes in *Xenopus* tumor with the different control tissues (TI-leg1-3 or NI-body1-3). X-axis shows M.value (log2 fold changes) in *Xenopus* tumor with the control tissue TI-leg1-3 (DEG4) and Y-axis shows M.value in *Xenopus* tumor with the control tissue NI-body1-3 (DEG8). (c) Expression pattern of DEG13 genes in *Xenopus* tumor and human cancer (bone tissue). X-axis shows M.value (log2 fold changes) in *Xenopus* tumor (DEG4) and Y-axis shows M.value in human cancer (DEG11). Red dots indicate genes that clearly exhibit differential gene expression pattern (for instance, increase in human cancer and decrease in *Xenopus* tumor). (d) Expression pattern of DEG14 genes in *Xenopus* tumor with the different control tissues (TI-leg1-3 or NI-body1-3). X-axis shows M.value (log2 fold changes) in *Xenopus* tumor with the control tissue TI-leg1-3 (DEG4) and Y-axis shows M.value in *Xenopus* tumor with the control tissue NI-body1-3 (DEG8). (e) Expression pattern of DEG14 genes in *Xenopus* tumor and human cancer (connective tissues etc.). X-axis shows M.value (log2 fold changes) in *Xenopus* tumor (DEG4) and Y-axis shows M.value in human cancer (DEG12).

Figure S4

a

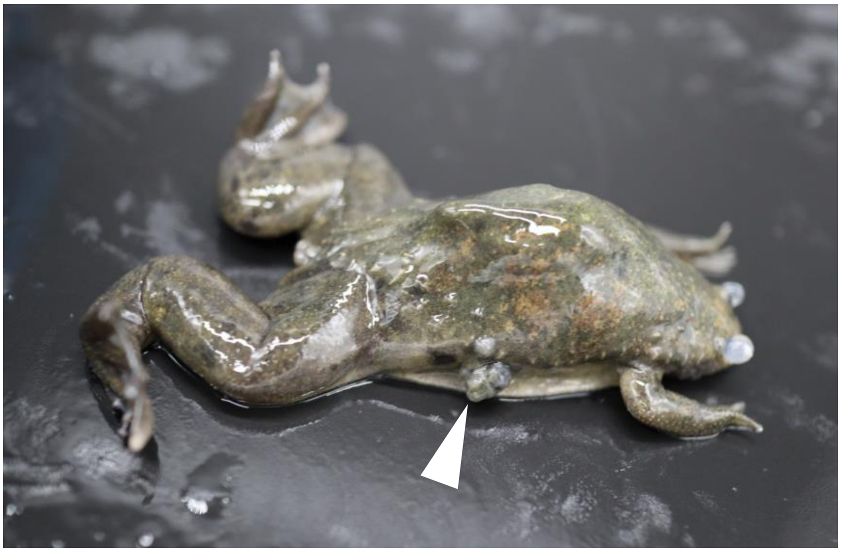

Figure S4

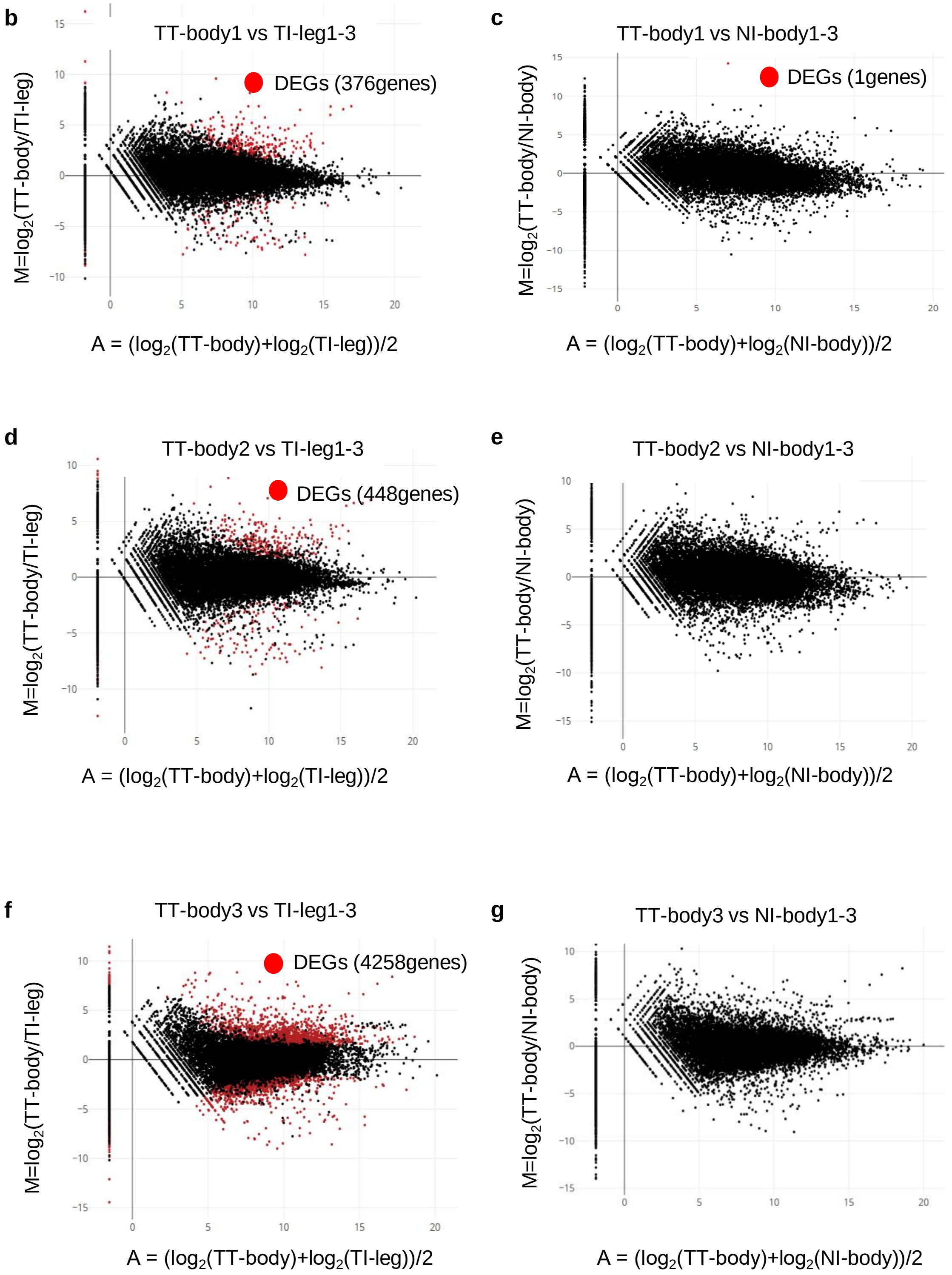

Fig. S4 DEGs by comparing gene expression pattern of TT-body1, 2 or 3 and TI-leg1-3 or NI-body1-3.

(a) External view of TT-body1 (NH-VIII-7). Arrowhead indicates tumor tissue. (b-c) DEGs of TT-body1 vs TI-leg1-3 (b) or NI-body1-3 (c). (d-e) DEGs of TT-body2 vs TI-leg1-3 (d) or NI-body1-3 (e). (f-g) DEGs of TT-body3 vs TI-leg1-3 (f) or NI-body1-3 (g).

Figure S5

a

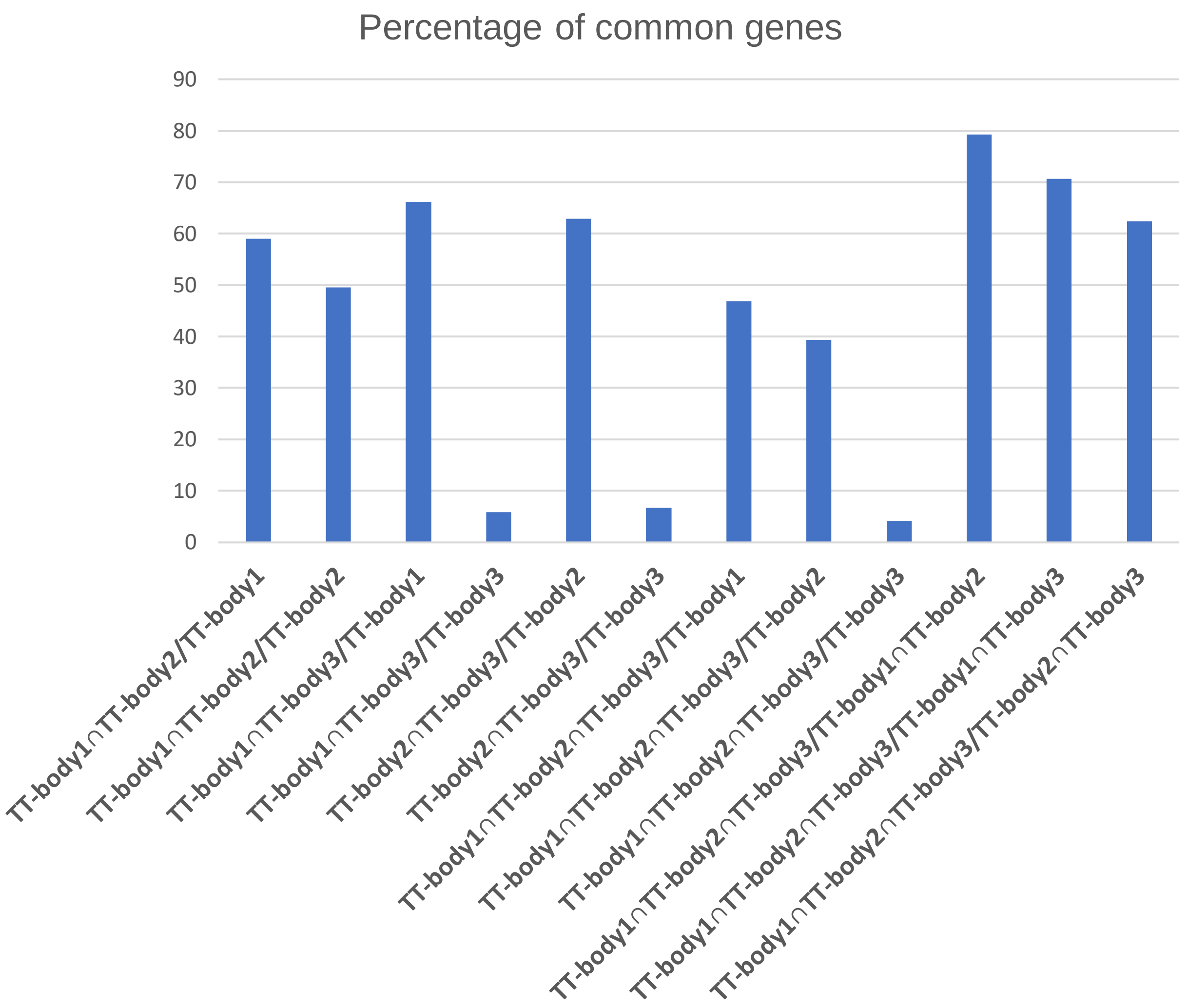
Percentage of common genes
90
80
70
60
50
40
30
20
10
0
TT-body1∩TT-body2/TT-body1
TT-body1∩TT-body2/TT-body2
TT-body1∩TT-body3/TT-body1
TT-body1∩TT-body3/TT-body3
TT-body2∩TT-body3/TT-body2
TT-body2∩TT-body3/TT-body3
TT-body1∩TT-body2∩TT-body3/TT-body1
TT-body1∩TT-body2∩TT-body3/TT-body2
TT-body1∩TT-body2∩TT-body3/TT-body3
TT-body1∩TT-body2∩TT-body3/TT-body1∩TT-body2
TT-body1∩TT-body2∩TT-body3/TT-body1∩TT-body3
TT-body1∩TT-body2∩TT-body3/TT-body2∩TT-body3

b

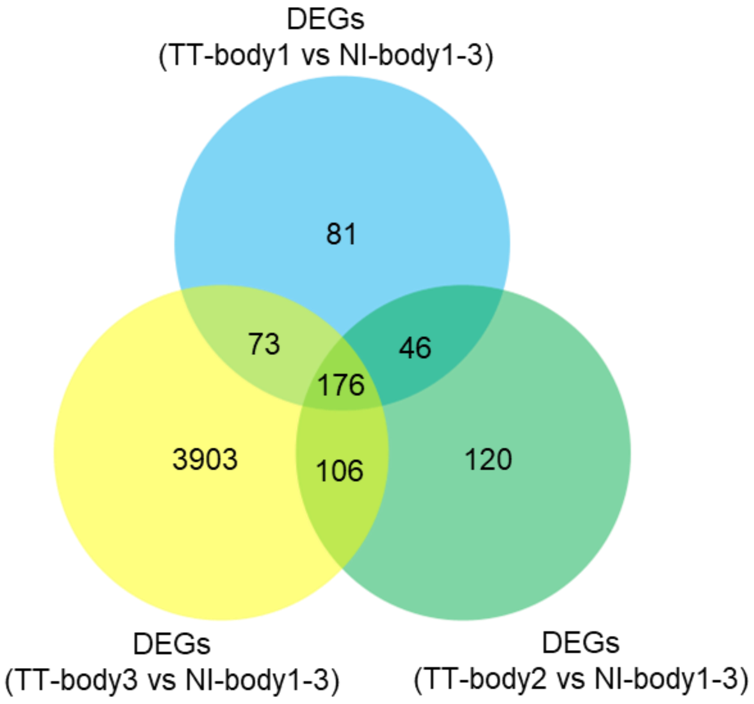
DEGs
(TT-body1 vs NI-body1-3)
81
73
46
176
3903
106
120
DEGs
(TT-body3 vs NI-body1-3)
DEGs
(TT-body2 vs NI-body1-3)

Fig. S5 Common genes of DEGs among TT-body1, 2 and 3.

These DEGs were extracted by comparing each TT-body and NI-body1-3. (a) Percentage of common genes among DEGs for each sample of TT-body. For instance, the leftmost bar shows the proportion of DEGs in TT-body1 that are common to TT-body1 and TT-body2. (b) Venn diagram among DEGs of TT-body1, TT-body2 and TT-body3. The number of common genes is indicated.

Figure S6

a

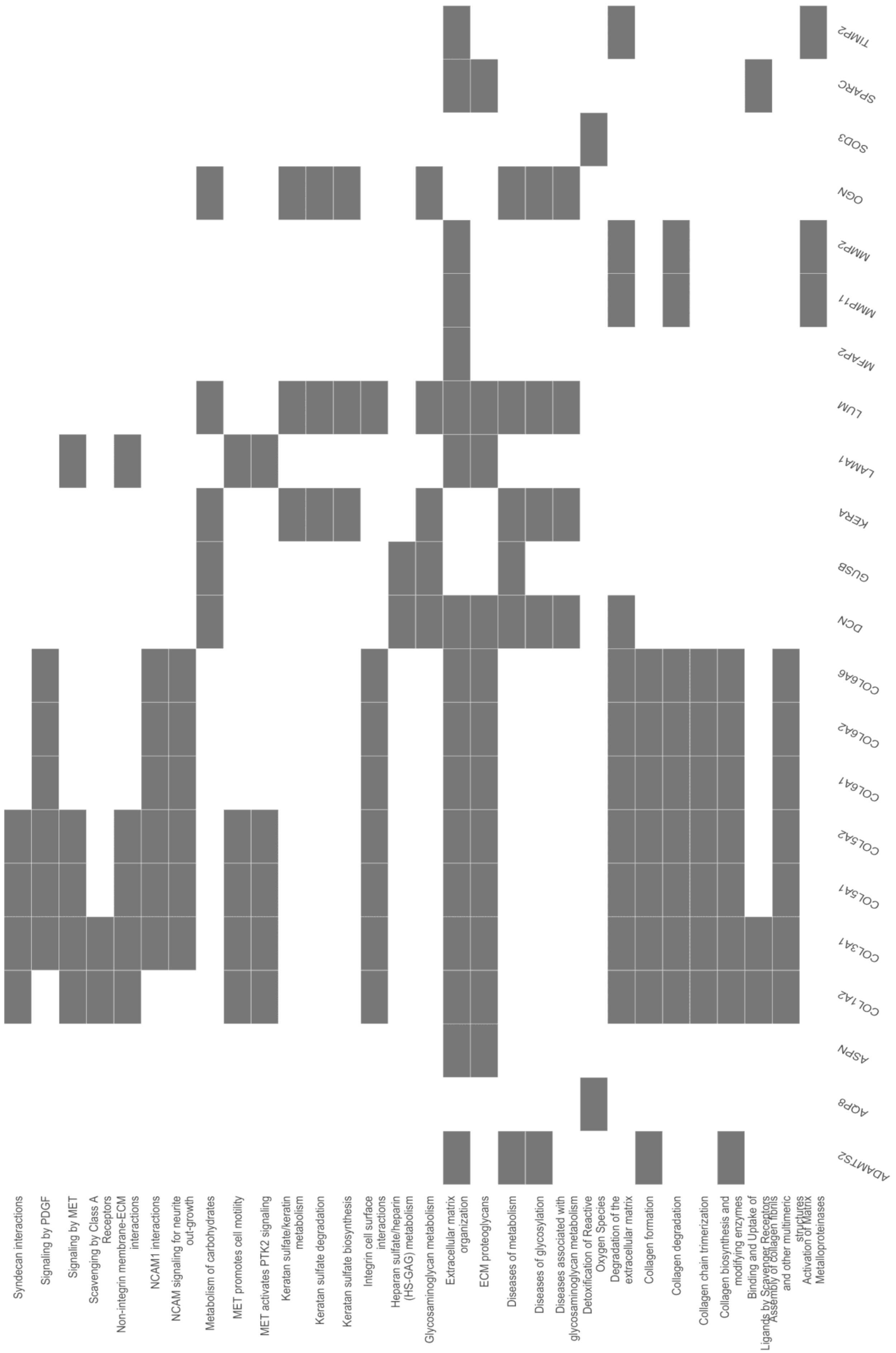

TIMP2
SPARC
SOD3
OGN
MMP2
MMP11
MFAP2
LUM
LAMA1
KERA
GUSB
DCN
COL6A6
COL6A2
COL6A1
COL5A2
COL5A1
COL3A1
COL1A2
ASPN
AQP8
ADAMTS2
Syndecan interactions
Signaling by PDGF
Signaling by MET
Scavenging by Class A Receptors
Non-integrin membrane-ECM interactions
NCAM1 interactions
NCAM signaling for neurite out-growth
Metabolism of carbohydrates
MET promotes cell motility
MET activates PTK2 signaling
Keratan sulfate/keratin metabolism
Keratan sulfate degradation
Keratan sulfate biosynthesis
Integrin cell surface interactions
Heparan sulfate/heparin (HS-GAG) metabolism
Glycosaminoglycan metabolism
Extracellular matrix organization
ECM proteoglycans
Diseases of metabolism
Diseases of glycosylation
Diseases associated with glycosaminoglycan metabolism
Detoxification of Reactive Oxygen Species
Degradation of the extracellular matrix
Collagen formation
Collagen degradation
Collagen chain trimerization
Collagen biosynthesis and modifying enzymes
Binding and Uptake of Ligands by Scavenger Receptors
Assembly of collagen fibrils and other multimeric structures
Activation of Matrix Metalloproteinases

Figure S6

b

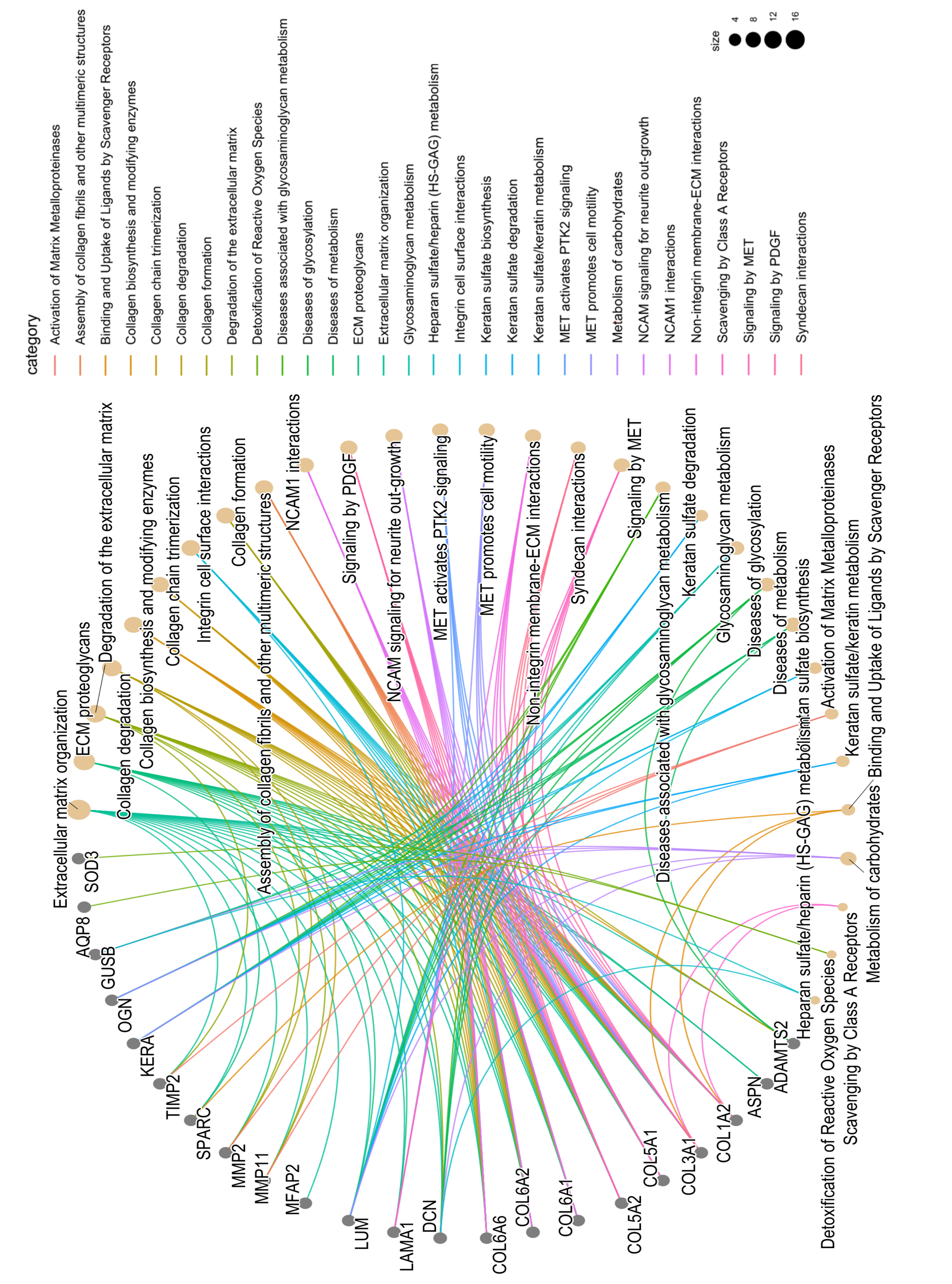
category
Activation of Matrix Metalloproteinases
Assembly of collagen fibrils and other multimeric structures
Binding and Uptake of Ligands by Scavenger Receptors
Collagen biosynthesis and modifying enzymes
Collagen chain trimerization
Collagen degradation
Collagen formation
Degradation of the extracellular matrix
Detoxification of Reactive Oxygen Species
Diseases associated with glycosaminoglycan metabolism
Diseases of glycosylation
Diseases of metabolism
ECM proteoglycans
Extracellular matrix organization
Glycosaminoglycan metabolism
Heparan sulfate/heparin (HS-GAG) metabolism
Integrin cell surface interactions
Keratan sulfate biosynthesis
Keratan sulfate degradation
Keratan sulfate/keratin metabolism
MET activates PTK2 signaling
MET promotes cell motility
Metabolism of carbohydrates
NCAM signaling for neurite out-growth
NCAM1 interactions
Non-integrin membrane-ECM interactions
Scavenging by Class A Receptors
Signaling by MET
Signaling by PDGF
Syndecan interactions
size
4
8
12
16
AQP8
SOD3
GUSB
OGN
KERA
TIMP2
SPARC
MMP2
MMP11
MFAP2
LUM
LAMA1
DCN
COL6A6
COL6A2
COL6A1
COL5A2
COL5A1
COL3A1
COL1A2
ASPN
ADAMTS2

Fig. S6 Reactome pathway analysis of DEG10 genes.

(a) The functional classification of DEG10 genes. This indicates genes enriched for pathway IDs. (b) Gene-Concept Network of genes enriched for pathway IDs. The circle size indicates the number of genes in the pathway ID.

Figure S7

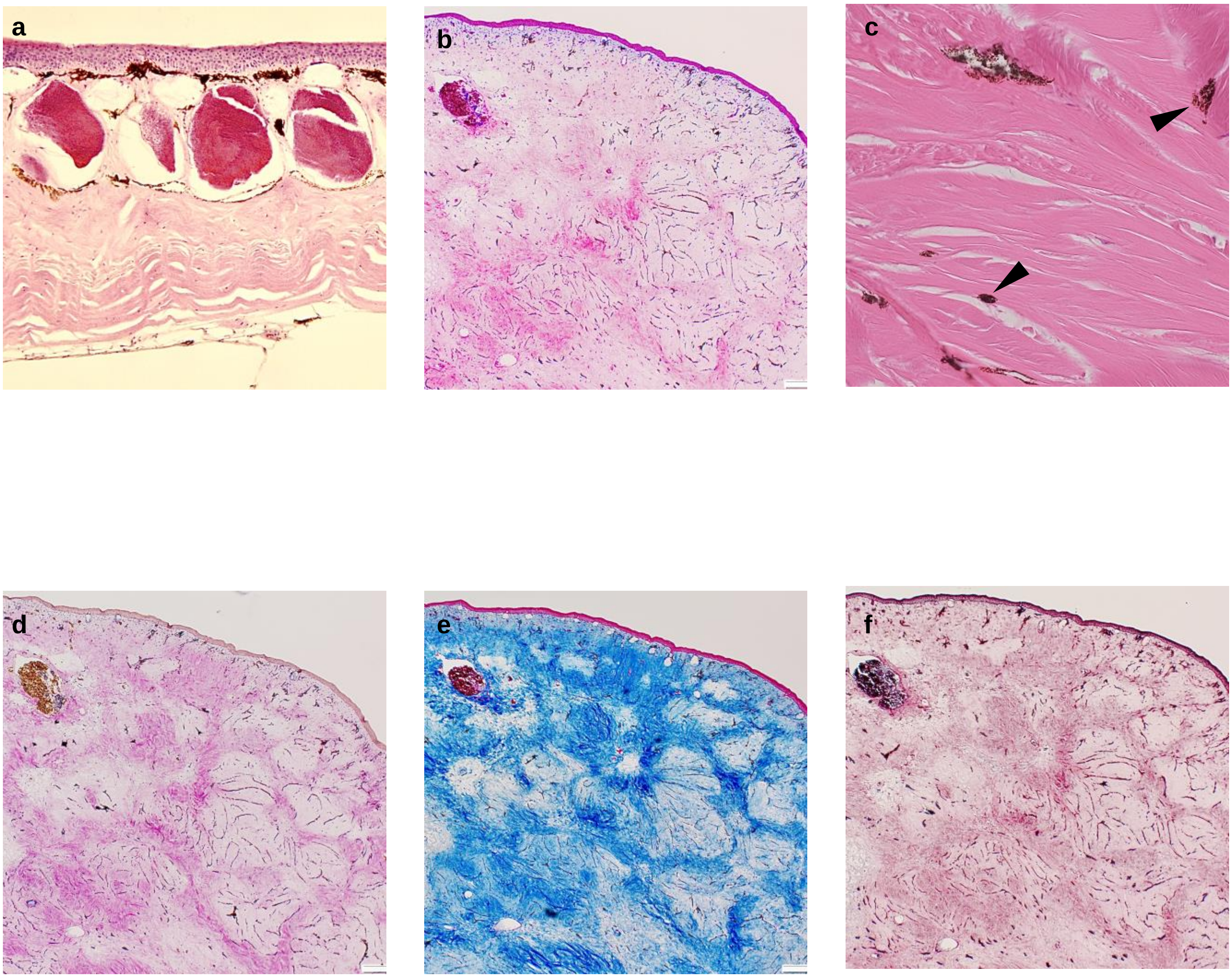

Fig. S7 Collagen staining in *Xenopus* tumor.

(a-c) HE staining of normal skin tissue (a), which is the same tissue used in Fig. 1, and tumor tissue (b-c). Tumor tissue with unclear borders was observed under the epidermis (b). The tumor has expansion of the dermis with compression and disappearance/atrophy of structures such as granules and glands, located beneath the epidermis. At high magnification (c) of the dermis in the tumor tissue, meandering fibers were observed. Pigments were observed between the fibers (arrowhead). (d) Elastica van Gieson staining of the tumor. The dermis was stained red, indicating that the fibers consists of collagen. (e) Azan staining of the tumor. Blue staining was observed, indicating that collagen fibers include type I collagen. (f) Silver staining of the tumor. This fiber was also stained by silver, indicating that the fibers include type III collagen.

Figure S8

a

Adrenal gland

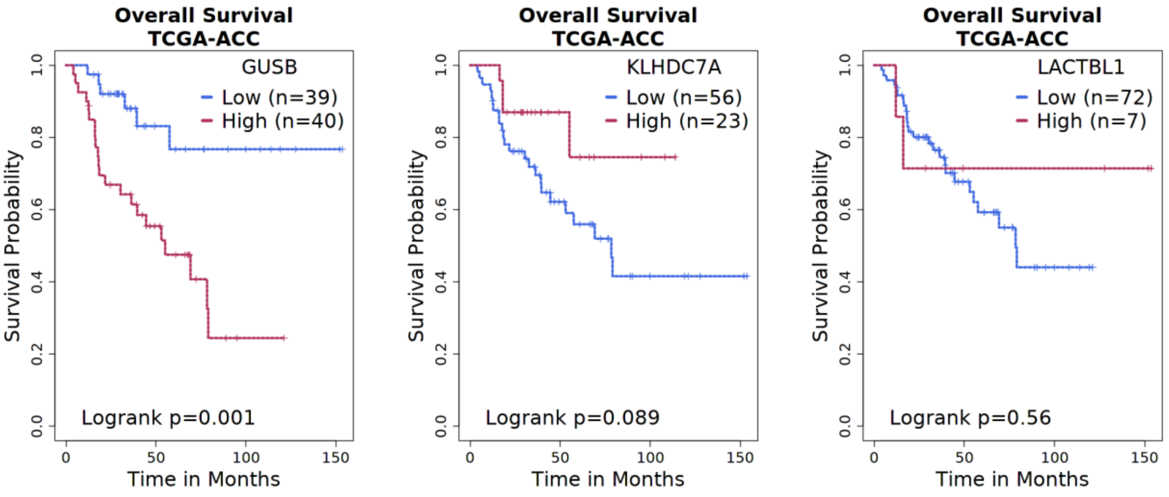

b

Bladder

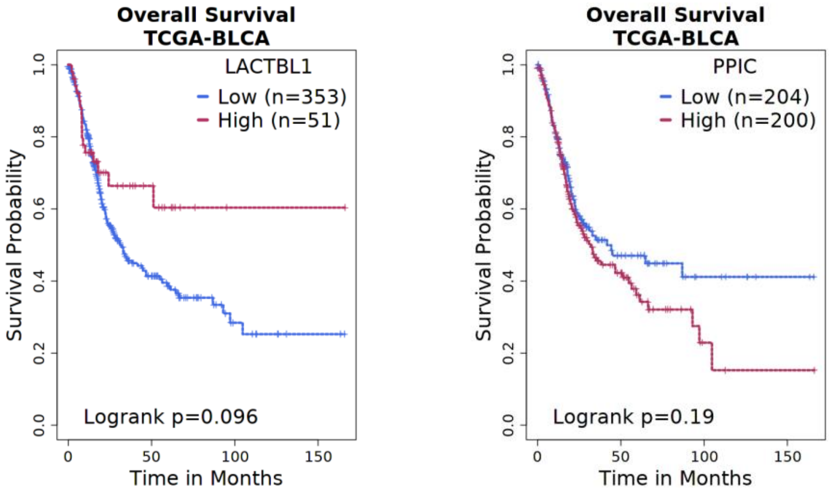

c

Cervix uteri

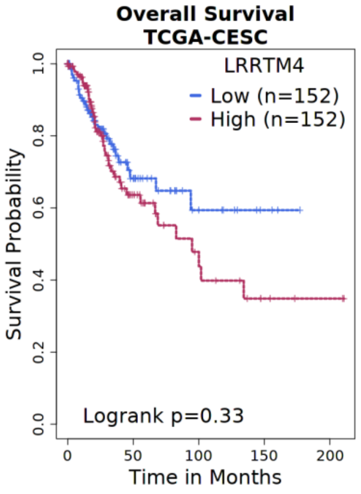

d

Kidney

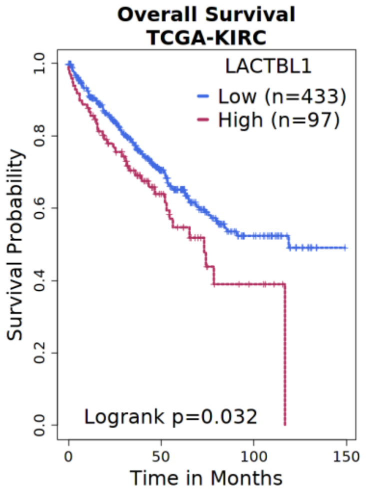

Figure S8

e

Kidney

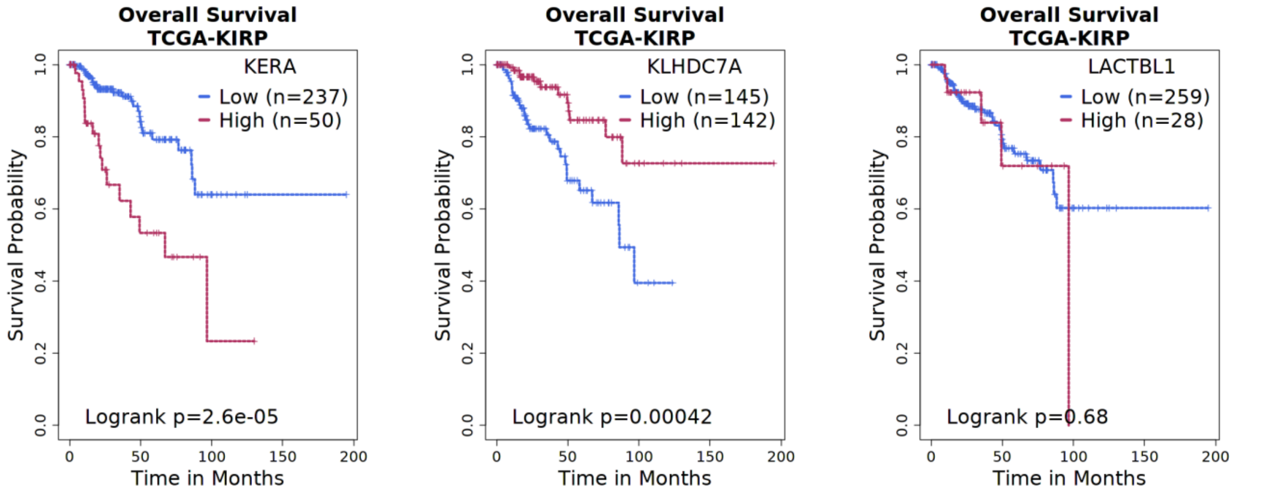

f

Brain

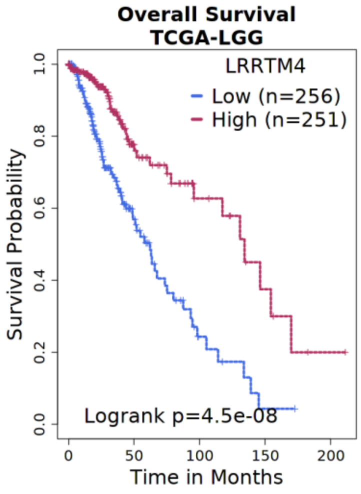

g

Pancreas

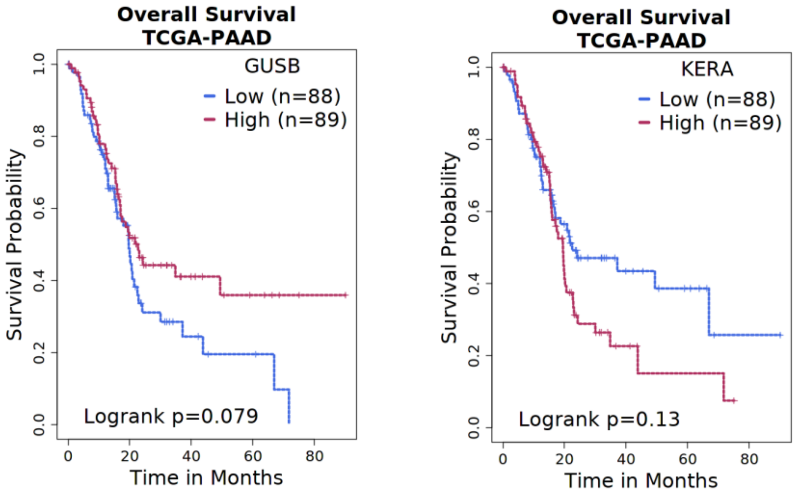

h

Bones, joints and articular cartilage of limbs, Colon, Connective, subcutaneous and other soft tissues, Corpus uteri, Kidney, Meninges, Other and unspecified male genital organs, Other and unspecified parts of tongue, Ovary, Peripheral nerves and autonomic nervous system, Retroperitoneum and peritoneum, Stomach, Uterus, NOS

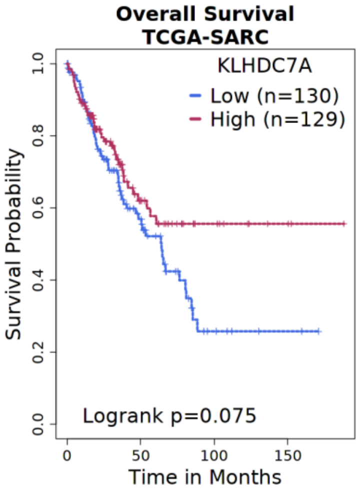

Figure S8

i

Colon, Connective, subcutaneous and other soft tissues, Rectosigmoid junction, Rectum, Unknown

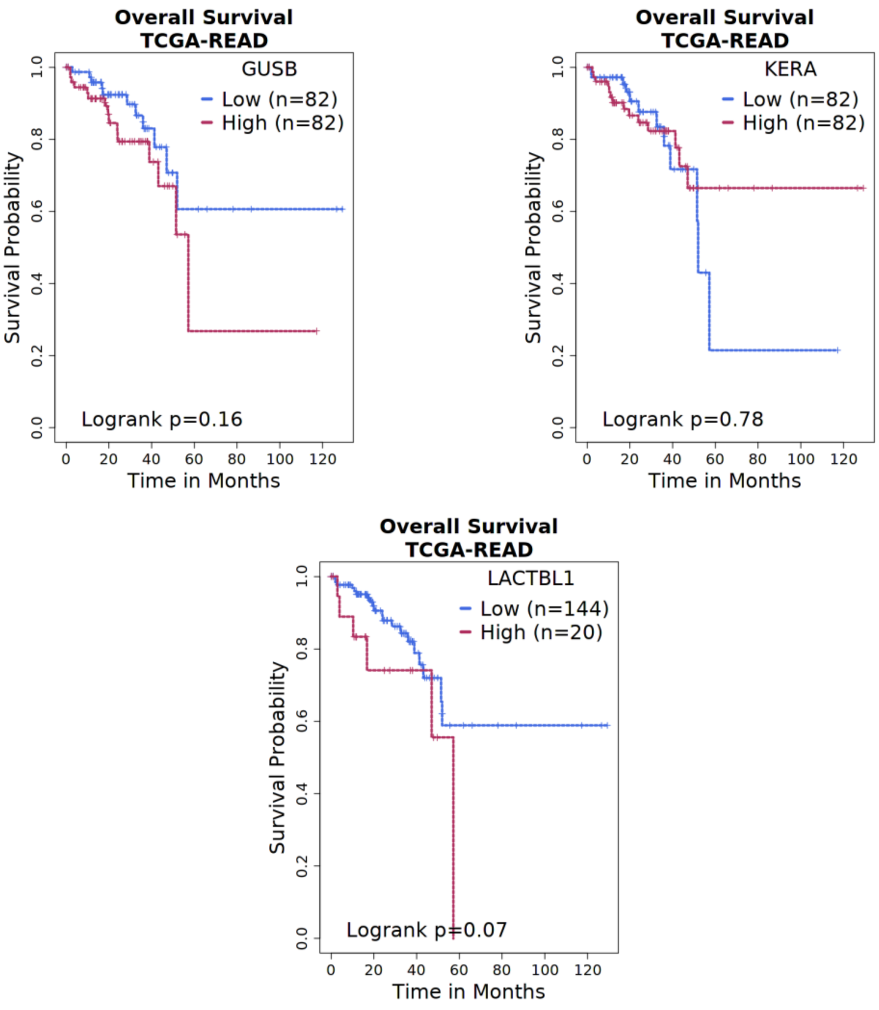

j

Stomach

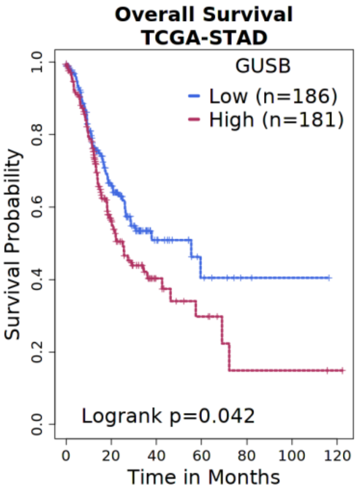

k

Uterus, NOS

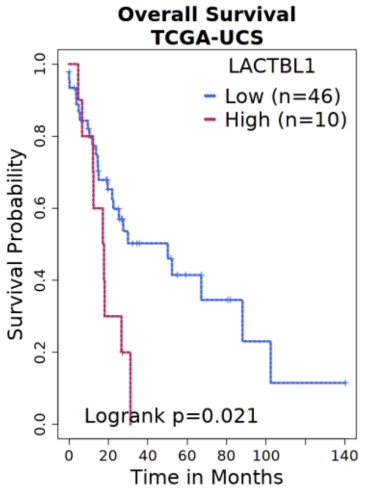

Figure S8

l

Corpus uteri, Uterus, NOS

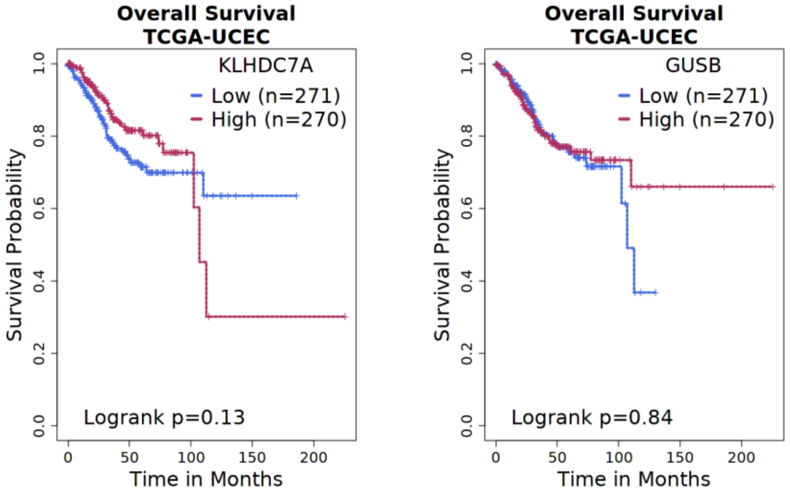

m

Eye and adnexa

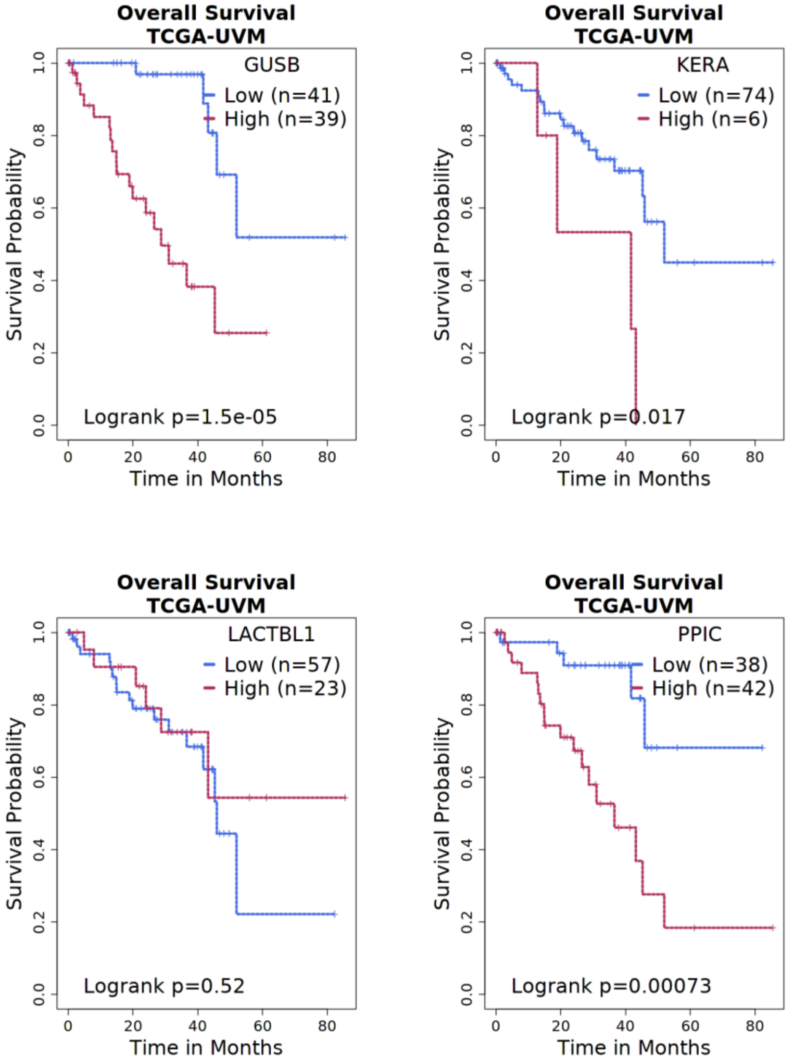

Fig. S8 Differences in survival rates depending on whether genes in DEG10 are highly or lowly expressed.

Kaplan-Meier analysis of genes in DEG10. Genes that were not well reported regarding cancer were analyzed. Genes that showed about > 0.2 difference in the survival probability between high- and low-expressed populations was shown. (a-m) is results of survival probability in the different cancer project of TCGA: TCGA-ACC (Adrenal gland) (a), TCGA-BLCA (Bladder) (b), TCGA-CESC (Cervix uteri) (c), TCGA-KIRC (Kidney) (d), TCGA-KIRP (Kidney) (e), TCGA-LGG (Brain) (f), TCGA-PAAD (Pancreas) (g), TCGA-SARC (Bones etc.) (h), TCGA-READ (Colon etc.) (i), TCGA-STAD (Stomach) (j), TCGA-UCS (Uterus, NOS) (k), TCGA-UCEC (Corpus uteri, Uterus, NOS) (l), TCGA-UVM (Eye and adnexa) (m).
